# Examining the Left‐Right Divide Through the Lens of a Global Crisis: Ideological Differences and Their Implications for Responses to the COVID‐19 Pandemic

**DOI:** 10.1111/pops.12740

**Published:** 2021-05-05

**Authors:** Benjamin Coe Ruisch, Courtney Moore, Javier Granados Samayoa, Shelby Boggs, Jesse Ladanyi, Russell Fazio

**Affiliations:** ^1^ The Ohio State University

**Keywords:** COVID‐19, ideology, individual differences, politics

## Abstract

The COVID‐19 disease pandemic is one of the most pressing global health issues of our time. Nevertheless, responses to the pandemic exhibit a stark ideological divide, with political conservatives (versus liberals/progressives) expressing less concern about the virus and less behavioral compliance with efforts to combat it. Drawing from decades of research on the psychological underpinnings of ideology, in four studies (total *N* = 4441) we examine the factors that contribute to the ideological gap in pandemic response—across domains including personality (e.g., empathic concern), attitudes (e.g., trust in science), information (e.g., COVID‐19 knowledge), vulnerability (e.g., preexisting medical conditions), demographics (e.g., education, income) and environment (e.g., local COVID‐19 infection rates). This work provides insight into the most proximal drivers of this ideological divide and also helps fill a long‐standing theoretical and empirical gap regarding how these various ideological differences shape responses to complex real‐world sociopolitical events. Among our key findings are the central role of attitude‐ and belief‐related factors (e.g., trust in science and trust in Trump)—and the relatively weaker influence of several domain‐general personality factors (empathic concern, disgust sensitivity, conspiratorial ideation). We conclude by considering possible explanations for these findings and their broader implications for our understanding of political ideology.

**Highlights**

- Stark ideological differences exist across a wide range of attitudinal and behavioral indices of pandemic response, with more conservative individuals reliably exhibiting less concern about the virus. These findings illustrate the extent to which the pandemic has become politicized.
- A range of factors contribute to this ideological gap in pandemic response, but some are substantially more important than others.
- Several factors that have received attention in public and academic discourse about the pandemic appear to contribute little, if at all, to the ideological divide. These include news following, scientific literacy, perceived social norms, and knowledge about the virus.
- The most critical factors appear to be trust in scientists and trust in Trump, which further highlights the politicization of COVID‐19 and, importantly, the antagonistic nature of these two beliefs. Efforts to change and, especially, disentangle these two attitudes have the potential to be effective interventions.

The divide between the political left and right is increasingly important—and increasingly acrimonious (e.g., Pew Research Center, 2014, 2016, 2017a; Reiljan, 2020). Indeed, in many nations, including the United States, the liberal‐conservative divide is among the most contentious divisions in modern society, eliciting levels of explicit antipathy that can even outpace differences based on race, religion, or social class (Pew Research Center, 2016, 2017a, 2017b). This animosity between those on the left and right stems largely from the deep‐seated differences in values, worldviews, and culture that characterize those of opposing ideologies (Wetherell, Brandt, & Reyna, 2013).

The past 70 years have witnessed much research aimed at understanding the nature and extent of the differences between individuals on the left and right. This research has identified numerous domains and dimensions of ideological differences, suggesting that liberals and conservatives differ in many aspects of everyday life and behavior (e.g., pastimes, jobs/careers; DellaPosta, Shi, & Macy, 2015), beliefs (e.g., trust in science, (Nadelson et al., 2014), personality (e.g., conscientiousness; Gerber, Huber, Doherty, Dowling, & Ha, 2010), emotional experience (e.g., sensitivity to disgust; Inbar, Pizarro, & Bloom, 2009), psychological motivations (e.g., needs for certainty; Jost, 2017), basic cognition (e.g., confidence; Ruisch & Stern, 2021), and even physiology (Oxley et al., 2008; Ruisch, Anderson, Inbar, & Pizarro, 2020).

Despite this ever‐growing list of ideological differences, however, we know relatively little about whether, how, and to what degree each of these differences shape real‐world political cognition and behavior, such as responses to salient sociopolitical events. That is, although we know that individuals of opposing ideologies *descriptively* differ in many ways, we understand less about the downstream implications of these differences for how liberals and conservatives interpret and respond to the political world.

Such questions, of course, are critical from a practical perspective: Political behavior such as voting, protest, and political violence have a profound impact on the lives and livelihoods of people the world over. But these questions are also critical from a theoretical perspective. Understanding the implications of these ideological differences is necessary for the construction of coherent and predictive models of political behavior. For example, which ideological differences do and do not shape real‐world political cognition and behavior? How powerful are these effects? Which factors are most proximal in predicting political outcomes? How do these factors interrelate and interact with one another?

The theoretical importance of these questions is underscored by recent developments in the field. In recent years, many independent lines of research have begun to challenge the dominant narrative that liberals and conservatives differ widely in their basic psychological traits and motivations (e.g., Choma & Hodson, 2017; Crawford, 2017; Wetherell et al., 2013)—criticisms that have been redoubled following recent failures to replicate some of the cornerstone findings of this area of research (e.g., Bakker, Schumacher, Gothreau, & Arceneaux, 2020).

Thus, in addition to facilitating the construction of more comprehensive models of political behavior (e.g., understanding the strength and primacy of these personality, belief, demographic, and environmental differences in predicting behavior), examining the downstream consequences of ideological differences will also contribute to resolving a number of outstanding empirical and theoretical issues in the field. In particular, this approach can help identify which previously documented effects do—and which do not—truly represent meaningful ideological differences that are consequential for political cognition and behavior.

## The COVID‐19 Pandemic

The COVID‐19 pandemic that began in late 2019 devastated nations around the world, claiming the lives of hundreds of thousands of people and bringing about negative economic repercussions that will likely be felt for years to come. Despite these profound and wide‐ranging impacts, however, attitudes and behavior towards the COVID‐19 pandemic in the United States, like many things in modern American society, have been characterized by a stark ideological divide (e.g., Pew Research Center, 2020). Those on the political left (progressives/liberals) express more concern about COVID‐19 and greater support for the social‐distancing guidelines that have been recommended by health experts and many government leaders. Those on the political right (conservatives), conversely, express less concern about the pandemic and lower support for measures intended to limit the spread of the virus.

The COVID‐19 pandemic is one of the most pressing global health crises of our time, and, as such, understanding these ideological differences in attitudes and behavior toward the pandemic is important in its own right. However, responses to the pandemic are also well suited to answering the theoretical and empirical questions raised above. In particular, the nature of the pandemic intersects with a number of broad ideological differences that have been documented in past research and/or predicted by theoretical models of ideology. These include attitudes towards science, scientific literacy, disgust sensitivity, perceived vulnerability to disease, empathic concern, and conspiratorial ideation, among others.

Perhaps most critically, the pandemic is a complex issue about which our understanding and capacity to successfully manage have been uncertain and rapidly changing. Unlike the issues examined in laboratory studies, the pandemic is a multifaceted subject that requires complex “trade‐offs” between different concerns—for example, pitting physical safety against economic security, ideological identity—along with cues emanating from relevant political elites—against dispositional traits like sensitivity to disgust. These factors make the pandemic an ideal lens through which to examine the influence of the various personality, belief, demographic, and environmental differences that characterize the ideological divide.

## The Present Research

In this research, we aim to help fill the theoretical and empirical gaps outlined above. To do so, we conducted a series of four studies (total *N* = 4441) in which we tested a range of theoretically relevant predictors to determine whether and how each contributes to the ideological divide in pandemic response. These factors include a range of attitudinal (e.g., trust in science), personality (e.g., disgust sensitivity), knowledge (e.g., accurate information about COVID‐19), demographic (e.g., education), and environmental predictors (e.g., COVID‐19 prevalence).

We used a multifaceted measure of pandemic response, examining several distinct components of attitudes toward the pandemic and self‐reported behavior (e.g., social distancing, handwashing). Further, we helped overcome the limitations of self‐report measures of behavior by utilizing an innovative, behaviorally oriented measure of social distancing: virtual graphical scenarios that ask participants to position themselves in realistic real‐world situations. These measures were developed in our lab (Fazio, Ruisch, Moore, Granados Samayoa, Boggs, & Ladanyi, 2021a) to provide a more behavioral measure of social distancing that relies on concrete, “in‐the‐moment” decisions about virtually simulated behaviors. These include situations such as distancing oneself from an oncoming walker, placing oneself on a crowded beach, and separating individuals waiting in line (demo measures available at http://psychvault.org/social‐distancing‐measures/). Critically, these virtual measures of social distancing have been shown to prospectively predict whether an individual subsequently contracts COVID‐19—and even to outpredict self‐reported social distancing (Fazio, Ruisch, Moore, Granados Samayoa, Boggs, & Ladanyi, 2021b). Thus, these measures provide an important additional index of pandemic response with consequential real‐world outcomes.

In these studies, we recruited large samples to provide stable and accurate estimates of effect sizes (Schönbrodt & Perugini, 2013). These samples provide us with sufficient statistical power to detect small effect sizes and to examine interactions and interrelations between individual factors. For example, our combined sample size provides us with 99% power to detect effects as small as *r* = .07.^1^

## Method

We tested a range of potential explanations for the ideological divide in responses to COVID‐19, deriving our predictions from past theory and research on ideological differences. These studies were conducted at four time points ranging from the height of the pandemic (April 22, 2020), when most citizens were under strict lockdown orders, to later (June 9, 2020), when some states had begun to reopen but social distancing was still required.

Given the large number of measures included in these studies, we utilized a “planned missing” design (Graham, Taylor, Olchowski, & Cumsille, 2006) in which different subsets of participants completed different groups of measures to minimize participant fatigue.^2^ All participants completed a common “core” survey that included our 21‐item multifaceted measure of pandemic response, with six items assessing attitudes towards the pandemic (e.g., worry about COVID‐19, perceptions that the threat is exaggerated, support for social distancing), five items assessing self‐reported behavior (e.g., social distancing, mask wearing, handwashing), and our 10 virtual social‐distancing measures. The core survey also included demographic questions and our measure of political orientation (“Please select the scale point that best reflects your political orientation,” assessed on a 7‐point scale from “Extremely liberal” to “Extremely conservative; Jost, 2006).

Our individual difference measures were grouped and randomized between participants based on theoretical and empirical relatedness (Table 1). Measures with conceptual overlap, theoretical relations, and/or established empirical associations were administered to the same subsample of participants. In this way, the variables most likely to “overlap” in the variance that they explained (e.g., disgust sensitivity and perceived vulnerability to disease) were grouped together so that we could adjudicate between them to identify the most proximal and powerful predictors of attitudes and behavior. Detailed information about all measures and procedures is contained in the online supporting information.

**Table 1:** Design for All Studies

Design Table												
Measures by Study and Sample												
	Study 1				Study 2				Study 3	Study 4		
	1A^a^	1B	1C	1D	2A	2B	2C	2D	3A	4A	4B	4C
Sample size	121	128	131	118	460	446	450	467	230	442	458	434
Personality‐type traits												
Conspiratorial ideation	X				X					X		
Empathic concern			X				X				X	
Disgust sensitivity				X				X				X
Attitudes and beliefs												
Trust in state governors						X				X		
Trust in president Trump						X				X		
Trust in science		X				X				X		
Perceived social norms									X	X	X	X
Knowledge and information												
News source ideological slant	X				X				X	X	X	X
Scientific literacy		X				X						
Objective COVID‐19 knowledge	X				X	X	X	X	X	X	X	X
Demographic factors												
Income	X	X	X	X	X	X	X	X	X	X	X	X
Education	X	X	X	X	X	X	X	X	X	X	X	X
Race/Ethnicity	X	X	X	X	X	X	X	X	X	X	X	X
Religion					X	X	X	X				
Religiosity					X	X	X	X				
Vulnerability												
Age	X	X	X	X	X	X	X	X	X	X	X	X
Negative economic consequences	X	X	X	X	X	X	X	X		X	X	X
Preexisting conditions					X	X	X	X	X	X	X	X
Suffering/Negative impact												
Perceived vulnerability to disease				X				X				X
Contracting COVID‐19	X	X	X	X	X	X	X	X		X	X	X
Personal job loss	X	X	X	X	X	X	X	X	X	X	X	X
Familial job loss	X	X	X	X	X	X	X	X				
Environmental factors												
Local COVID‐19 prevalence	X	X	X	X	X	X	X	X	X	X	X	X
PersonsPerSquareMile	X	X	X	X	X	X	X	X	X	X	X	X
County‐level conservatism	X	X	X	X	X	X	X	X	X	X	X	X
Racial diversity	X	X	X	X	X	X	X	X	X	X	X	X
Median income	X	X	X	X	X	X	X	X	X	X	X	X
Age: % 65 and over	X	X	X	X	X	X	X	X	X	X	X	X
Income inequality	X	X	X	X	X	X	X	X	X	X	X	X
Governor party	X	X	X	X	X	X	X	X	X	X	X	X
Local reopening status					X	X	X	X	X	X	X	X

^a^ Letters denote independent participant subsamples.

Finally, we also examined a wide range of potentially relevant macrolevel and environmental factors. To do so, we collected information about participants' geographic location via IP‐address‐based geolocation in combination with their provided zip code. We then mapped this information onto data collected from various databases (e.g., the U.S. Census Bureau; see the online supporting information) to obtain measures such as city‐ and county‐level indices of COVID‐19 prevalence and death rates, population density, and age distribution.

Several of these studies included identical measures. Given that study did not reliably moderate these effects, for brevity, to maximize statistical power, and to provide a more accurate estimate of effect sizes (Braver, Thoemmes, & Rosenthal, 2014), we present our results by measure, collapsing across individual studies. However, none of our conclusions are substantively altered if these studies are analyzed independently.

### Participants

We recruited participants from Amazon's Mechanical Turk (MTurk; see Buhrmester, Kwang, & Gosling, 2011). We chose this participant pool for several reasons. First, the nature of the pandemic presented several ethical, practical, and generalizability‐related challenges for selecting a participant sample. Given the risk of transmitting the virus, face‐to‐face interaction (e.g., for survey administration) was both ethically indefensible and in violation of local laws and ordinances. Second, although not a representative sample of the U.S. population, participants from Mechanical Turk are considerably more demographically diverse than the college‐student samples used in most psychological research (Paolacci & Chandler, 2014). Critically, this diversity extends to political ideology as well: Although MTurk participants skew somewhat more politically liberal on average, they are considerably more politically diverse than college‐student samples (Berinsky, Huber, & Lenz, 2012).

Also critical in our decision to use this pool was that MTurk workers are geographically diverse—in our studies, coming from all 50 U.S. states. This factor was particularly important given the unequal geographic distribution of the virus, as well as the substantial state‐to‐state and community‐to‐community variability in social‐distancing regulations and other measures taken to combat the virus. Given these advantages, and the fact that MTurk participants perform similarly to non‐MTurk samples across many tasks and measures (Berinsky et al., 2012), including surveys on political attitudes and related belief systems (Clifford, Jewell, & Waggoner, 2015), we judged this sample as offering an appropriate and illustrative test of our research questions. (Demographic characteristics available in Table 2).

Based on recent best‐practice recommendations for working with MTurk samples (e.g., Robinson, Rosenzweig, Moss, & Litman, 2019), we took several steps to ensure good data quality, including the use of an attention/comprehension check. We excluded participants who failed this check (11.8% of participants, leaving a total sample of 3885). However, none of our results are substantively altered if these participants are included in analyses.

### Structure and Presentation of Results

Because of the large number of measures, hypotheses, and relevant background literatures, for the sake of conciseness and clarity, we have structured this article somewhat unconventionally. The remainder of the article is organized into subsections, which are structured as “miniarticles” consisting of any necessary relevant theoretical background, methods, results, and a brief discussion. In these sections, we examine our various predictor measures to determine whether each factor (1) predicts responses to the pandemic and (2) is associated with political ideology. Finally, we examine (3) whether that factor contributes to the ideological gap in pandemic response. To assess this latter question, we conducted mediation analyses estimating the indirect effect of each factor in statistically accounting for the relation between ideology and pandemic response (Table 3).

**Table 2:** Demographic Information for All Studies

Demographic Information												

	Study 1				Study 2				Study 3	Study 4		
	1A^a^	1B	1C	1D	2A	2B	2C	2D	3A	4A	4B	4C
Sample size	121	128	131	118	460	446	450	467	230	442	458	434
Age												
Mean age	37.19	38.86	37.56	36.31	38.98	39.48	38.42	38.93	37.23	37.61	37.92	38.59
SD age	10.68	11.29	11.08	10.41	12.23	12.74	12.53	12.53	11.29	11.99	11.62	12.30
Min. age	18	20	20	21	18	18	19	18	18	19	18	18
Max. age	72	68	78	71	77	89	76	76	72	79	74	70
Gender												
% Women	65.29	63.28	55.73	72.03	53.48	50.90	55.33	51.18	65.22	54.07	51.09	58.29
% Men	34.71	35.16	43.51	27.97	45.43	48.43	44.22	48.18	33.48	45.48	48.03	41.24
Race												
% White	82.64	75.00	72.52	71.19	75.87	71.97	74.67	71.95		75.57	75.98	75.35
% Black	12.40	13.28	14.50	16.10	10.87	13.00	12.00	13.28		11.76	11.35	12.67
% Hispanic	1.65	7.03	8.40	8.47	5.87	6.28	6.44	6.85		7.01	5.68	5.76
% Asian	5.79	7.03	6.11	4.24	8.91	9.42	8.00	10.06		8.60	7.42	8.06
Income and education												
Median income	$50‐75K	$50‐75K	$50‐75K	$50‐75K	$50‐75K	$50‐75K	$50‐75K	$50‐75K	$50‐75K	$50‐75K	$50‐75K	$50‐75K
Median education	College Grad.	College Grad.	College Grad.	College Grad.	College Grad.	College Grad.	College Grad.	College Grad.	College Grad.	College Grad.	College Grad.	College Grad.
Political orientation												
% Liberal	46.28	46.09	51.91	48.31	47.61	47.98	56.89	54.39	51.74	46.83	47.82	50.23
% Moderate	14.05	12.50	12.98	13.56	19.13	19.51	14.22	16.06	13.04	20.36	15.94	13.36
% Conservative	39.67	40.62	35.11	38.14	33.04	32.51	28.89	29.55	35.22	32.81	36.24	35.94

^a^ Letters denote independent participant subsamples.

**Table 3:** Primary Analyses

Summary of Primary Analyses				

Category	Factor	Ideology β/*r*	Pandemic Response β/*r*	Indirect Effect β
Personality‐type traits	1. Conspiratorial ideation	.17	−.17	−.02
	2. Empathic concern	−.12	.30	−.03
	3. Disgust sensitivity	.16	.23	
Attitudes and beliefs	4. Trust in state governors	−.08	.24	.02
	5. Trust in president Trump	.59	−.32	−.14
	6. Trust in science	−.46	.44	−.18
	7. Perceived social norms	.14	(.05)	
Knowledge and information	8. News source ideological slant	.43	.18	−.04
	9. Scientific literacy	.19	(−.05)	
	10. Objective COVID‐19 knowledge	−.26	.16	−.03
Demographic factors	11. Income	.05	(.01)	
	12. Education	(−.01)	.08	
	13. Race (non‐White vs. White)	.03	−.07	
	14. Religion (non‐Christian vs. Christian)	.35	−.10	
	15. Religiosity	.37	−.10	
Vulnerability	16. Age	.06	.08	
	17. Preexisting conditions	.06	.16	
	18. Perceived vulnerability to disease	(−.05)	.37	
Suffering/Negative impact	19. Contracting COVID‐19	.03	−.04	
	20. Negative economic consequences	.09	.09	
	21. Personal job loss	(−.01)	.04	
	22. Familial job loss	(−.01)	(.01)	
Environmental factors	23. COVID prevalence in community	−.08	.08	−.005
	24. Population density	−.05	.04	
	25. County‐level conservatism	.14	−.08	−.006
	26. Racial diversity	−.06	.05	.002
	27. Median income	(−.02)	.05	
	28. Age distribution (% 65 and over)	−.02	(−.02)	
	29. Income inequality	−.06	−.03	
	30. Governor party (Dem. vs. Rep.)	.05	−.06	
	31. Local reopening status	.06	.10	.006

Note.

Columns indicate (1) the relation between the target variable and ideology (greater numbers indicate greater conservatism); (2) the relation between each factor and pandemic response (greater numbers indicate greater concern about the virus); and (3) whether the factor statistically mediated the relation between ideology and COVID‐19 response. If so, the estimated indirect effect is provided. Nonsignificant effects/relations are in parentheses.

These factors—over 30 in total—have been loosely organized into the superordinate categories shown in the following list. The sequential numbers in the list correspond to the later sections of the article and the online supporting information.

1. *Personality Trait‐Type Factors*: conspiratorial ideation, empathic concern, disgust sensitivity.
2. *Attitude and Belief Factors*: trust in state governors, trust in the president, attitudes towards science, and perceived social norms.
3. *Knowledge and Information Factors*: news sources, scientific literacy, objective knowledge about COVID‐19.
4. *Demographic Factors*: income, education, race, religion, and religiosity.
5. *Vulnerability Factors*: age, preexisting conditions, perceived vulnerability to disease.
6. *Suffering/Negative‐Impact Factors*: contracting COVID‐19, negative economic consequences, personal and/or familial job loss.
7. *Environmental Factors*: Objective COVID‐19 prevalence in one's local community; county‐level measures of population density, percent conservative versus liberal, racial diversity, median income, age distribution, income inequality; whether one's local community has reopened and/or relaxed social‐distancing guidelines; and the political party of one's state governor.

## The Ideological Gap in Pandemic Response

As anticipated, we observed stark ideological differences in pandemic response, with more conservative individuals exhibiting less concern about the virus. The strength of this association differed somewhat across measures, with some items (e.g., perceiving that the severity of the pandemic is exaggerated, *r* = .38) showing relatively strong associations with ideology, and others (e.g., worry about personally contracting the virus, *r* = −.08) showing relatively smaller, albeit statistically significant, associations. Critically, however, these ideological differences emerged for attitudes, self‐reported behavior, and virtual behavior—and, in fact, were significant on all 21 of the individual items that comprised our dependent measure (all *p*s < .02). These findings provide important evidence that these ideological differences are not limited to any one domain—e.g., attitudes but not behavior—but emerge across different dimensions of pandemic response. Given this consistency, for all analyses we simply use the 21‐item composite “pandemic response” measure, which we treat as a general index of concern about the virus. Using this composite measure, the relation between ideology and pandemic response was *r*/*β* = −.29 (*t*(3879) = 18.81, *p* < .001).

## [1] PERSONALITY TRAIT‐TYPE FACTORS

As discussed above, liberals and conservatives are argued to differ on a wide variety of personality‐related dimensions. We selected three factors that we viewed as most likely to shape responses to the pandemic.

### Conspiratorial Ideation

Several conspiracy theories have arisen regarding the pandemic, suggesting that the virus was created by humans and/or that its severity is intentionally exaggerated to manipulate the public (Mitchell & Oliphant, 2020). Endorsement of these conspiracy theories relates to less concern about the virus (Imhoff & Lamberty, 2020). Given research suggesting that conservatives may have a greater propensity toward conspiratorial thinking (Lamberty, Hellmann, & Oeberst, 2018), we examined the role of conspiratorial ideation in driving the ideological gap in pandemic response. We assessed both general conspiratorial ideation (Brotherton, French, & Pickering, 2013) and endorsement of two conspiracy theories specifically related to the pandemic: the belief that the virus is human‐made, and that its severity is exaggerated to mislead the public.

### Disgust Sensitivity

Disgust plays an important role in disease prevention, motivating avoidance of potentially pathogenic objects and individuals (Schaller, 2011). Accordingly, sensitivity to disgust should motivate greater concern about COVID‐19—a prediction supported by recent research (Shook, Sevi, Lee, Oosterhoff, & Fitzgerald, 2020). Given that liberals and conservatives have been shown to differ in disgust sensitivity (Inbar et al., 2009), we assessed this factor. To do so, we administered two widely used measures of disgust sensitivity, the Pathogen Disgust subscale of the Three‐Doman Disgust Scale (Tybur, Lieberman, & Griskevicius, 2009; Study 1) and the Contamination subscale of the Disgust Scale‐Revised (Olatunji et al., 2007; Studies 2 & 4).

### Empathic Concern

Empathic concern for others may motivate people to socially distance (Pfattheicher, Nockur, Böhm, Sassenrath, & Petersen, 2020). Some research has suggested that liberals and conservatives may differ in the degree and/or scope of their empathic concern—for example, with conservatives exhibiting greater empathy for ingroup members and liberals being more likely to exhibit empathy across group boundaries (Waytz, Iyer, Young, Haidt, & Graham, 2019). We therefore wished to examine whether these differences help explain the ideological divide in pandemic response. To do so, we measured both general empathic concern (using the compassionate love for humanity scale; Sprecher & Fehr, 2005; and the perspective‐taking and empathic concern subscales of the Interpersonal Reactivity Index; Davis, 1980) as well as a novel measure of compassion for individuals afflicted by COVID‐19.

### Results

#### COVID Response

The relations between these measures and responses to the pandemic generally supported our predictions. Both general conspiratorial ideation (*β* = −.17, *t*(1021) = 5.37, *p* < .001) and endorsement of COVID‐19‐related conspiracy theories (*β* = −.32, *t*(1330) = 12.32, *p* < .001) predicted less concern about the virus. Disgust sensitivity, conversely, predicted greater concern about the virus (*β* = .23, *t*(1016) = 7.35, *p* < .001), as did empathy—both general empathy (*β* = .30, *t*(1037) = 10.26, *p* < .001) and empathy towards the victims of COVID‐19 (*β* = .55, *t*(1037) = 21.05, *p* < .001). All associations remained significant when controlling for political ideology (*p*s < .002).

#### Ideological Differences

Consistent with some past work, we found that conspiratorial ideation was significantly higher among more conservative individuals (*β* = .17, *t*(1020) = 5.59, *p* < .001). However, these ideological differences accounted for only a relatively small portion (6.8%) of the ideological gap in pandemic response (indirect effect: *β* = −.02, 95% CI[−.04, −.009]). We also found that conservatives expressed considerably greater endorsement of *COVID‐19‐specific* conspiracy theories (*β* = .40, *t*(440) = 9.28, *p* < .001). Interestingly, however, this association was only partially accounted for by general conspiratorial ideation (indirect effect: *β* = .15, 95% CI[.09, .21]).

We found a similar pattern of results regarding empathy. More conservative individuals scored slightly, although significantly, lower in empathic concern (*β* = −.12, *t*(1037) = 4.00, *p* < .001). These differences accounted for a significant—although again relatively modest (10.3%)—portion of the ideological gap in responses to the pandemic (indirect effect: *β* = −.03, 95% CI[−.05, −.02]). We also found that conservatives expressed considerably less empathy for sufferers of COVID‐19 (*β* = −.23, *t*(1037) = 7.68, *p* < .001). Paralleling the results with conspiratorial ideation, however, this association was only partially accounted for by general ideological differences in empathy (indirect effect: *β* = −.06, 95% CI[−.09, −.03]).

Thus, the above factors mediated the ideological gap as hypothesized—although their explanatory power was somewhat modest. The pattern of effects regarding disgust sensitivity, however, diverged more substantially from the predictions of past theory and research. As in past work, we found that conservatives scored higher in sensitivity to disgust (*β* = .16, *t*(1014) = 5.30, *p* < .001). However, given that conservatives exhibited less concern about COVID‐19, disgust sensitivity, unsurprisingly, did not explain the ideological gap—and, in fact, statistically adjusting for disgust sensitivity only made these ideological differences emerge more starkly (without disgust covariate: *β* = −.32; with disgust: *β* = −.36; *p*s < .001).

### Discussion

Our findings regarding the existence and direction of liberal‐conservative differences in the above personality trait‐type factors largely replicated previous research. However, the role that these factors played in driving the ideological gap in pandemic response differed somewhat from the predictions of past theory and research. Conspiratorial ideation and empathic concern differed as predicted between liberals and conservatives: Liberals were higher in empathic concern, while conservatives were higher in conspiratorial ideation. Interestingly, however, these factors accounted for a relatively modest portion of the ideological gap in responses to the pandemic (6.8% and 10.3%, respectively). Further, they explained only a portion of the variance in closely conceptually related outcomes—compassion for COVID‐19 victims (26%) and endorsement of conspiracy theories about the virus (37.5%).

More striking still was the pattern of effects with disgust sensitivity. As in past research, conservatives scored higher in sensitivity to disgust than did liberals. And yet, the observed ideological differences in disgust sensitivity—despite their clear theoretical relevance for responses to a global disease pandemic—did not lead conservatives to exhibit greater concern about the pandemic; rather, despite their greater sensitivity to disgust, conservatives expressed substantially less concern about the pandemic.

Critical to note, of course, is that some of these personality trait‐type ideological differences *did* appear to matter. However, their role in explaining the liberal‐conservative gap in pandemic response was somewhat modest. These findings seem to suggest that at least some of the personality‐trait‐type ideological differences that have been documented in past research may play a relatively indirect role in shaping cognition and behavior regarding real‐world sociopolitical events, at least under the current circumstances. Even these highly relevant personality‐related factors explained a relatively small portion of the liberal‐conservative differences in pandemic response. In other words, the reason that liberals and conservatives differ in their compassion for sufferers of COVID‐19 and their endorsement of COVID‐related conspiracy theories—as well as their responses to the pandemic more generally—is not solely, and perhaps not even primarily, because they differ in lower‐level, domain‐general psychological traits and motivations. Rather, these ideological differences in pandemic response appear in large part to stem from elsewhere. Next, we turn to the role of attitude and beliefs in explaining these differences.

## [2] ATTITUDE AND BELIEF FACTORS

Based on past theory and research, we identified several attitude and belief‐related factors that might help explain the ideological gap in pandemic response.

### Trust in Government

The coronavirus pandemic had little direct precedent in modern American history, and the information landscape, particularly early in the pandemic, was complex and rapidly changing. The novelty and complexity of pandemic, coupled with the highly polarized American political context, is likely to have rendered Americans' views particularly attuned to “elite cues,” or the messages emanating from salient leaders such as government officials (Levendusky, 2010). However, given the sharply differing responses of different leaders and experts (e.g., the president, medical experts, state governors), the particular cues that a given individual followed is likely to have been starkly moderated by their degree of (dis)trust in these various officials (Zaller, 1992). We assessed trust in (1) the federal government, (2) President Trump, and (3) state governors.

### Attitudes Towards Science

Information about COVID‐19 comes largely from scientific research. However, the established ideological differences in trust in science (e.g., Pew Research Center, 2019) may have moderated liberals' and conservatives' receptivity to these messages (Zaller, 1992). We therefore examined this factor as well. Our measure comprised both belief in science/the scientific method (e.g., as a valid path to knowledge; Farias, Newheiser, Kahane, & de Toledo, 2013) and trust in scientists (e.g., that scientists are objective and do not intentionally misreport findings; Nadelson et al., 2014).

### Perceived Social Norms

Social norms exert powerful effects on human behavior, including compliance with behavioral directives (Cialdini & Goldstein, 2004). Given the ideological divide in responses to the pandemic, we predicted that liberals and conservatives might differ in their perceptions of the normativity of social distancing. We assessed norm perceptions using both a self‐report measure (“To what extent do you think that Americans in general are currently following social distancing guidelines?”; Study 4) and a more in‐depth measure in which participants completed our virtual distancing measures as if they were “the average American” (Study 3).

### Results and Discussion

#### COVID Response

As anticipated, our three measures of trust in government related very differently to concern about the virus. General trust in the federal government did not predict responses to the pandemic (*β* = −.06, *p* = .06), perhaps reflecting the divergent messages of different government leaders. Trust in Donald Trump, however, predicted less concern about the virus (*β* = −.32, *t*(885) = 10.07, *p* < .001)—an association that remained significant even after adjusting for participants' own political ideology (*β* = −.23, *t*(884) = 5.90, *p* < .001). Conversely, trust in state governors predicted greater concern about the virus (*β* = .24, *t*(885) = 7.34, *p* < .001), even after adjusting for participants' own ideology, the political party of their state governor, and the own‐ versus‐governor‐ideology interaction (*β* = .28, *t*(879) = 8.56, *p* < .001). This association may reflect the fact that state governors generally advocated stricter guidelines to address the virus (Burns, Martin, & Haberman, 2020).

Attitudes towards science were a powerful predictor of responses to the pandemic (*β* = .44, *t*(1014) = 15.61, *p* < .001). Perceived norms, conversely, were only weakly, if at all, related to pandemic response: The association with the self‐report norm item was very weak (*β* = .06, *t*(1332) = 2.04, *p* = .04), and the more in‐depth “respond‐as‐the‐average‐American” questions were completely unrelated to participants' own responses to the pandemic (*β* = .01, *p* = .86).

#### Ideological Differences

More conservative individuals expressed greater trust in the federal government (*β* = .36, *t*(1011) = 12.16, *p* < .001) and greater trust in Donald Trump (*β* = .59, *t*(885) = 21.76, *p* < .001). Liberals expressed slightly higher support for state governors (*β* = .08, *t*(885) = 2.49, *p* = .01). These differences in trust in state governors accounted for a statistically significant, although small, portion of the gap (indirect effect: *β* = .02, 95% CI[.005, .04]). Trust in Trump, conversely, accounted for a more substantial portion of this gap (indirect effect: *β* = −.14, 95% CI[−.19. −.08]). (However, given the high correlation between ideology and trust in Trump, *r* = .59, these results should be interpreted cautiously.)

As predicted, conservatism was also associated with less trust/belief in science (*β* = −.46, *t*(1013) = 16.53, *p* < .001). These ideological differences in attitudes towards science, in turn, accounted for a significant portion of the ideological gap—and in fact had a larger effect size than any previously examined factor (indirect effect: *β* = −.18, 95% CI[−.22, −.15]). This effect remained significant when adjusting for the other attitude‐ and belief‐related factors (indirect effect: *β* = −.13, 95% CI[−.23, −.05]).

The pattern of effects with perceived social norms were also intriguing. Norm perceptions did not explain the ideological gap. In fact, conservatives—despite personally social distancing less—believed that social distancing among Americans was *more* normative (*β* = .14, *t*(1560) = 5.73, *p* < .001). Indeed, both liberals and conservatives alike seemed to be relatively unaffected by what “Americans in general” were doing: As noted above, there was little correspondence between perceived norms and personal attitudes.

In sum, these attitude and belief factors accounted for a larger portion of the ideological gap and generally seemed to play a stronger role than the personality‐related factors examined above. Although social‐norm perceptions mattered little, both trust in Trump and, especially, attitudes towards science, appeared to be key, accounting for a substantial portion of the ideological gap in responses to the pandemic. Although our data do not allow us to decisively speak to the mechanism behind these effects, this pattern is consistent with the possibility that Americans' attitudes toward the pandemic were, in large part, adopted from salient political and expert elites: For conservatives, these messages emanated largely from Trump, while for liberals they came from medical and scientific experts.

## [3] KNOWLEDGE‐ AND INFORMATION‐RELATED FACTORS

Liberals and conservatives also differ in their knowledge about many aspects of the world—differences that stem in part from divergent news‐following behavior (Arceneaux, Johnson, & Cryderman, 2013). We focused on three factors that we viewed as especially relevant for pandemic response.

### News Sources

There are stark ideological differences in preferred news sources, with people tending to prefer news that is consistent with their own position on the ideological spectrum (Arceneaux et al., 2013). Given that liberal and conservative news sources differed substantially in their portrayal of the virus (e.g., Bird & Ritter, 2020), we examined the role of news sources. Participants indicated their primary news source by choosing from a range of liberal (e.g., MSNBC), conservative (Fox News), and moderate (ABC, CBS, NBC) options.

### Scientific Literacy

Beyond the aforementioned ideological differences in *attitudes* towards science, there is evidence that liberals and conservatives may differ in their scientific literacy (i.e., objective knowledge and understanding of scientific concepts; Carl, Cofnas, & of Menie, 2016). Given the role of scientific information in communication about the pandemic, we examined whether these differences helped explain the ideological gap. We measured scientific literacy with an 11‐item true/false quiz (e.g., “Electrons are smaller than atoms”; Miller, 1998).

### Objective COVID‐19 Knowledge

These ideological differences in scientific literacy and news following may also shape objective knowledge about COVID‐19. Given that knowledge about the virus shapes responses to the pandemic (Calvillo, Ross, Garcia, Smelter, & Rutchick, 2020), we also examined the role of COVID‐19 knowledge. We assessed knowledge with a 13‐item true/false measure (e.g., “Regularly rinsing your nose with saline will protect you against COVID‐19 / the coronavirus”).

### Results and Discussion

#### COVID Response

Having a more politically liberal (versus more conservative) primary news source was associated with greater concern about the virus (*β* = .23, *t*(1265) = 8.29, *p* < .001)—an association that remained significant after adjusting for participants' own ideology (*β* = .16, *t*(1262) = 5.45, *p* < .001). In exploratory analyses, we separated out the relative influence of individual liberal and conservative news sources. Here we found that following Fox News was uniquely powerful in predicting responses to the virus, with the association between following Fox News as a primary news source (*β* = −.12) being nearly double the average association (*β* = .07) of the three more liberal news sources (MSBC, CNN, NPR).

Similarly, objective knowledge predicted greater concern about the virus (*β* = .16, *t*(3506) = 9.47, *p* < .001). Scientific literacy, surprisingly, was not a significant predictor of responses to the pandemic (*β* = .05, *p* = .20).

#### Ideological Differences

As anticipated, participants' ideology was associated with the ideological slant of their primary news source (*β* = .35, *t*(1263) = 13.06, *p* < .001), such that people tended to favor ideologically consistent news. These ideological differences in news sources, in turn, accounted for a modest but statistically significant portion of the ideological gap (indirect effect: *β* = −.06, 95% CI[−.08, −.04]).

More liberal individuals scored higher in scientific literacy (*β* = .19, *t*(571) = 4.69, *p* < .001). However, because scientific literacy was not related to responses to the pandemic, this difference did not explain the ideological gap (indirect effect: *β* = .0001, 95% CI[−.01, .01]). Liberals also scored higher in objective knowledge about the virus (*β* = −.26, *t*(3503) = 15.87, *p* < .001), which accounted for a significant, although again relatively small, portion of the ideological gap (indirect effect: *β* = −.025, 95% CI[−.03, −.02]).

On the whole, ideological differences in knowledge and information‐related factors appeared to explain a small but statistically significant portion of the ideological gap in pandemic response. Liberal‐conservative differences in news sources and objective knowledge about the pandemic—although not differences in scientific literacy—each accounted for a small portion of the variance. These results largely mirror those of the findings above, suggesting that although most individual dimensions of ideological difference do not independently explain a large portion of the gap, they each contribute a small amount of explanatory power.

## [4–7] DEMOGRAPHIC, VULNERABILITY, NEGATIVE IMPACT, AND ENVIRONMENTAL FACTORS

We also examined a number of other possible explanations for the ideological gap in the domains of demographic factors (income, education, race, religion, religiosity), vulnerability to COVID‐19 (age, preexisting conditions, perceived vulnerability to disease), and negative impacts (personally contracting the disease, job loss, and negative economic consequences). Further, we also explored a wide range of environmental predictors, including objective COVID‐19 prevalence in one's community, county‐level indices of population density, percent conservative (versus liberal), and racial diversity; whether the participant's local community had relaxed social‐distancing guidelines; state governor political party; and factors such as county‐level median income, income inequality, and age distribution. Given space limitations, we discuss only a few key findings here. Additional analyses are available in the online supporting information.

On the whole, demographic factors were a resounding failure to explain the ideological gap. Although liberals and conservatives differed in factors such as income, race, religion, and religiosity, after accounting for ideology, these same demographic factors generally did not reliably predict responses to the pandemic. In other words, despite the manifold ways that factors like income, race, and religion might be expected to shape one's life experiences and worldview, none of these differences had substantial predictive power for responses to the pandemic.

Similarly, there was little evidence that (real or perceived) vulnerability played a meaningful role in the ideological gap. If anything, conservatives were somewhat more vulnerable to the disease, being somewhat older (*β* = .08, *t*(3877) = 5.22, *p* < .001) and more likely to have preexisting medical conditions (*β* = .06, *t*(3376) = 3.22, *p* = .001). However, the association of pandemic response with age (*r* = .06) and preexisting conditions (*r* = .16) was relatively small, suggesting that these factors may exert only a weak influence on responses to the virus. Further, the only vulnerability factor with substantial predictive power for pandemic response, perceived vulnerability to disease (*β* = .37, *t*(1015) = 12.72, *p* < .001), did not—contrary to past theory and research—differ between liberals and conservatives (*β* = −.05, *p* = .15).

We also found little evidence that the gap was explained by ideological differences in suffering or negative impacts from the virus. Although experiencing negative economic consequences as a result of the pandemic (e.g., losing a job) was generally associated with greater concern about the virus, liberals and conservatives did not reliably differ in the degree to which they and their families had suffered economically. Further, more conservative individuals were actually somewhat more likely to have (or believe they had) contracted the virus (logistic regression: *B* = −.06, χ^2^(1) = 4.02, *p* = .045), further suggesting that conservatives' lower concern about the pandemic was not a result of having suffered fewer consequences.

Of the many environmental factors we examined, the two most robust predictors of responses to the pandemic were county‐level COVID‐19 infection/death rates, which predicted greater concern about the virus (*β* = .08, *t*(3882) = 5.11, *p* < .001), and county‐level conservatism/liberalism (indexed by the Republican/Democratic vote ratio in the 2016 U.S. presidential election), which predicted less concern (*β* = −.08, *t*(3883) = 4.94, *p* < .001). (Both factors remained significant predictors after adjusting for participants' political ideology.) Each of these factors, in turn, accounted for a very small, but statistically significant, portion of the ideological gap (infection/death rates’ indirect effect: *β* = −.005, 95% CI[−.008, −.002]; county‐level conservatism’s indirect effect: *β* = −.006, 95% CI[−.01, −.001]).

Thus, the majority of these factors appeared to play little role in driving the ideological gap in pandemic response. Demographic, vulnerability, and negative‐impact factors mattered little, if at all. Similarly, most of the environmental factors we examined also did not account for this gap. The possible exceptions to this trend were objective infection/death rates and county‐level conservatism, both of which accounted for a statistically significant portion of the ideological gap. Although the portion of the variance accounted for by these factors was small, given the inherent imprecision associated with such county‐level measures, these effects may be underestimated. Nevertheless, on the whole, ideological differences in demographic, vulnerability, negative impact, and environmental factors do not appear to be key drivers of the ideological gap in pandemic response.

More generally, although we observed ideological differences across many dimensions, these findings suggest that many of these differences may be descriptive only and do not play an important role in shaping real‐world cognition and behavior. Although these null results should be interpreted cautiously, they are nonetheless intriguing. Given the importance of many of these factors (e.g., race, religion, income) for people's lives and worldviews and/or their direct relevance to the pandemic (e.g., preexisting conditions, COVID‐19 deaths in one's local community), it is surprising that these ideological differences did not play a stronger role in shaping liberals' and conservatives' responses to the pandemic. If these differences do matter for political behavior, they appear to be easily superseded by other more proximal concerns and motivations.

### General Discussion

In attempting to provide a more comprehensive account of the factors underlying the ideological divide in pandemic response, this article has, necessarily, covered a great deal of conceptual ground. Indeed, the past 70 years of research has uncovered numerous potentially relevant domains of ideological differences. The findings here provide insight into whether and to what degree ideological differences in many of these factors—over 30 in total—explain the liberal‐conservative gap in pandemic response.

Despite the wide range of dimensions we investigated, several broad themes emerged from these findings, both regarding the ideological gap in COVID‐19 response specifically, as well as the broader theoretical questions regarding the nature of ideology that were raised in the introduction. Among the most intriguing and consistent findings was the failure of individual domains of ideological differences to account for large portions of the ideological gap in pandemic response. Indeed, several domains of ideological differences, such as differences in demographic, vulnerability, and negative‐impact factors explained little to none of the liberal‐conservative divide in responses to the pandemic. Environmental differences, similarly, appeared to explain only a small portion of the gap.

The personality‐trait‐type individual differences that we examined were an important exception to this general trend, having played a somewhat larger role in accounting for the ideological gap. However, their explanatory power was somewhat more modest than the literature would suggest. We replicated previously documented ideological differences in empathic concern, conspiratorial ideation, and disgust sensitivity, and each of these factors predicted responses to the pandemic as expected. Nonetheless, only two of these three differences—empathic concern and conspiratorial ideation—significantly mediated the ideological gap in pandemic response, and the portion of the variance they explained was relatively modest.

These findings might be interpreted as lending further weight to recent critiques arguing that the scope of ideological differences is more limited than is often presumed. However, it is critical to note that these differences did matter; it is simply that the impact of most *individual factors* was relatively modest. Considered collectively, these factors nonetheless may account for a substantial portion of the liberal‐conservative gap. Further, although we selected traits that we viewed as particularly theoretically relevant for pandemic response, there are numerous personality‐related factors (e.g., openness to experience, conscientiousness) that reliably differ between liberals and conservatives but that we did not examine here.

Nonetheless, it seems that there may be a kind of “bottleneck” limiting the impact of many of these ideological differences—such as those related to personality‐type traits like conspiratorial ideation and disgust sensitivity—in shaping real‐world political cognition and behavior. That is, although these factors were robust and theoretically consistent predictors of pandemic response, *ideological differences* in these same traits appeared to be far less impactful.

One possible explanation for these findings, consistent with other critiques (e.g., Taber & Young, 2013), is that some portion of the ideological differences observed on these measures do not represent “real” differences in the underlying traits of interest. For example, they may in part reflect self‐presentational differences between those on the left and right or other issues related to the self‐report nature of these measures. To assess this possibility, other researchers may wish to use analogous non‐self‐report measures of these same traits to examine the degree to which they mediate the ideological gap in pandemic response. For example, behavioral or physiological measures of disgust sensitivity—which often correlate only weakly, if at all, with self‐reports (e.g., Smith, Oxley, Hibbing, Alford, & Hibbing, 2011)—might provide further insight into why ideological differences in self‐reported disgust sensitivity appeared to play little role in shaping responses to the pandemic.

An alternative possibility is that the ideological differences we observed on these measures may in fact represent real differences—but some of these differences were nonetheless superseded by other more important or proximal beliefs and motivations. This explanation seems consistent with the pattern of results that we observed here. Differences that seem relatively more “distal” from ideology—e.g., domain‐general personality‐trait‐type differences like empathic concern and disgust sensitivity—appeared to matter little. Conversely, more focused, domain‐specific attitudes that are more closely related to ideological content—especially trust in Trump and trust in science—accounted for more of the ideological gap.

One important outstanding question is whether this pattern of effects—especially the greater predictive power of ideologically relevant beliefs/attitudes versus broader personality‐type traits—is unique to this sociopolitical event or whether it is a more general phenomenon. On one hand, the central role of attitudes towards science and trust in the president may to some degree be due to idiosyncratic features of the U.S. sociopolitical context and the COVID‐19 pandemic. Specifically, there are several factors that may have led to an increased reliance on elite cues—and, in particular, the messages emanating from two of the most salient authorities, Trump and scientific experts. As noted, the pandemic was a complex and largely unprecedented event—in many ways a “hard” issue (Carmines & Stimson, 1980) that citizens may have struggled to connect to existing values and belief systems (Gilens & Murakawa, 2002). They may have therefore instead simply “fallen back” on cues from copartisans.

This reliance on elite cues may also have been further exacerbated by the gradual emergence of the virus on the U.S. political stage. Typically speaking, such an immediate and consequential issue as a global pandemic—where citizens' objective health and welfare is at stake—should be expected to circumvent the heuristic processing that leads people to simply “toe the party line” and instead elicit deeper deliberation about the issue (Prior, Sood, & Khanna, 2015). Such a process may have allowed individual differences (e.g., in personality factors such as disgust sensitivity) to play a stronger role in shaping political cognition and behavior. Importantly, however, the polarized response to the virus among American political elites substantially preceded the point at which the virus became a highly salient threat to Americans (e.g., well before a national emergency was declared in the United States; Green, Edgerton, Naftel, Shoub, & Cranmer, 2020). The relatively lower salience and personal relevance for Americans at these early stages may have led to a greater reliance on elite cues (Gilens & Murakawa, 2002). Once these attitudes were formed, however, motivated cognition and interpretation of subsequent information may have reinforced them, even after the pandemic became an immediate threat to American lives and livelihoods. Collectively, these factors may have substantially increased the power of trust in Trump and attitudes towards science in explaining the ideological gap in pandemic response, perhaps “washing out” the influence of ideological differences in more domain‐general personality‐type traits. Whatever the cause, however, the current findings offer a particularly stark illustration of the power of elite cues in guiding public opinion. Unlike the (relatively inconsequential) self‐report survey measures on which cue‐following effects are typically observed, these findings suggest that elite cues can also play a powerful role in attitude formation for issues with profound and immediate consequences for personal welfare.

An alternative possibility, however, is that the relatively weaker role of ideological differences in more domain‐general traits may be a more general phenomenon. For example, it may be that these broader personality traits shape the ideology that an individual initially adopts (e.g., in young adulthood), but later they exert little direct influence on real‐world political cognition. Future research will be needed to understand whether the lower predictive power of these seemingly more distal traits generalizes to other contexts and issues or whether these traits might be stronger predictors of political behavior under other conditions (e.g., for less polarized or salient political issues).

A consideration of these results within the broader international context may also shed light on some of these questions. In particular, future research may wish to compare the pattern of effects that we observed in the American context with other nations where the elite response to the pandemic has been less polarized. One close comparison case appears to be that of Canada, where the political elite was largely unified in its response to the virus (Merkley, Bridgman, Loewen, Owen, Ruths, & Zhilin, 2020). Intriguingly, however, even in the absence of polarized elite cues, left‐right political ideology nonetheless predicted responses to the pandemic (Merkley et al., 2020), albeit more modestly than in the United States. This may offer some convergent support for our interpretation of our findings. Although elite cues from Trump and scientific experts may have been among the most powerful drivers of attitudes in the United States—substantially exacerbating the ideological gap—there nonetheless appears to be something fundamental about political ideology (e.g., deeper domain‐general ideological differences in personality) that also influences responses to the pandemic.

## Supporting information

Supplementary Material

## Footnotes

^1^
All data, syntax, and materials are available on the Open Science Framework (OSF) at https://osf.io/45huw.

^2^
With the exception of Study 3, in which we included only a limited number of measures, and all participants completed the same measures.
