# Supplementary material for: Examining the Left‐Right Divide Through the Lens of a Global Crisis: Ideological Differences and Their Implications for Responses to the COVID‐19 Pandemic
Source: Polit Psychol. 2021 May 5;42(5):795–816. doi: 10.1111/pops.12740 (PMC8242330; doi:10.1111/pops.12740)
Supplement: Supplementary file 1 — Supplementary Material [file POPS-42-795-s001.docx]

**Supplemental Materials**

**Notes on Methodology and Analyses**

The majority of our dependent measures were identical across studies. However, in some instances we adapted our dependent measures to reflect the changing situation with the pandemic (e.g., changing the question about quarantine adherence to the past tense after stay-at-home orders were lifted, dropping no-longer-relevant items). Most predictor measures were included in two or three of our four studies. When scales appeared in multiple studies, they were generally identical at all time points. However, in a few instances, we also shortened or revised predictor scales to reduce overall study length or improve scale reliability. All substantive changes are noted in the appropriate sections below. The exact order and wording of all measures can be found on the OSF site for this project: https://osf.io/45huw/?view_only=adb31de0719b41288a622825d4c5d01e.

Mediation models were tested using Andrew Hayes’ PROCESS macro for SPSS. We used Model 4 and 10,000 bootstrapped samples. In the main text, we report the standardized indirect effects for any significant mediation models, along with 95% confidence intervals.

Variable names are provided in brackets below. These names correspond to the data and syntax files on the OSF page.

**Dependent Measures**

**Attitude Measures**

The attitudes component of our composite pandemic response measure was comprised of the six items listed below.

*All Studies:*

[Worried] “Generally speaking, how worried are you that you personally will contract COVID-19 / the coronavirus?” (1 Not worried at all – 7 Extremely worried)

[Likely] “Generally speaking, how likely do you think it is that you personally will contract COVID-19 / the coronavirus?” (1 Very unlikely – 7 Extremely likely)

[COVIDExaggerated] “Do you believe the threat of COVID-19 / the coronavirus has been exaggerated?” (4-point scale from “Yes, I believe the threat has been greatly exaggerated” to “No, I believe the threat has not been conveyed strongly enough”)

[EconVsCOVID] “Authorities have closed businesses and recommended strict "social-distancing" to keep people safe from COVID-19. However, many experts predict that the economy will suffer because of these regulations. In your personal opinion, how should authorities weigh these two concerns?” (6-point scale from “Authorities should ONLY focus on protecting people from COVID-19 / the coronavirus, regardless of how much the economy will suffer.” to “Authorities should ONLY focus on protecting the economy, regardless of how many people will suffer from COVID-19 / the coronavirus.”)

*Studies 2, 3, and 4 only:*

[SupportOpposeRule] “Do you support or oppose the guideline to engage in social distancing?”(-3 Strongly Oppose to +3 Strongly Support)

*Studies 2 and 3 only*:

[COVIDConcern] “How concerned are you about the spread of COVID-19 / the coronavirus?” (“1 Not concerned at all” to “5 Very concerned”)

**Self-Reported Behavior Measures**

The self-reported behavior component of our composite pandemic response measure was comprised of the five items listed below.

*All Studies*:

[HowOftenWashBefore] “How often are you now washing your hands or using hand sanitizer during a typical day compared to before the pandemic?”(7-point scale from “1 The same amount as before the pandemic” to “7 Much more than before the pandemic”)

[UseFaceMask] “How regularly do you wear a face mask or face covering any time you leave your home?”(6-point scale from “Never/Do not have one” to “All of the time”)

[SelfQuarantine] In Studies 1-3, participants were asked “Generally speaking, how strictly have you personally been following the "social distancing" recommendations of the government and CDC (for example, staying home except for absolute necessities, having no contact with other people outside your household, etc.)” In Study 4, to reflect recent changes to the social-distancing guidelines in the U.S., participants were asked “Up to a few weeks ago, most people were under “shelter-in-place” orders (for example, staying home except for absolute necessities, having no contact with other people outside your household, etc.). During that time, how strictly did you follow these recommendations?” Both questions were assessed on a 7-point scale from “1 I did not follow these recommendations at all” to “7 I followed these recommendations very strictly.”

*Studies 1 and 2 only*:

[ContactYesterday] “Not counting the members of your household, approximately how many people did you have "close contact" with yesterday? ("close contact" means being closer than 6 feet away, even if it was only briefly)”(7-options: “zero”, “one”, “two”, “3-5”, “5-10”, “10-20”, “More than 20”)

*Studies 3 and 4 only*:

[SelfDistancing] “Generally speaking, how strictly have you personally been following the "social distancing" recommendations of the government and CDC to maintain a distance of six feet or more from others?”(7-point scale from “1 I’m not following these recommendations at all” to “7 I’m following these recommendations very strictly”

**Virtual Social Distancing Measures**

The virtual social distancing component of our composite pandemic response measure was comprised of the ten items described below. Question text and screenshots of each item are provided. The interactive version of each measure can be found at <http://psychvault.org/social-distancing-measures/>

[TwoFriendsWoods] “Imagine you are out for a walk with a friend. What is the minimum distance you would feel comfortable having between the two of you?” This item was measured on an 11-point slider scale, corresponding to images depicting different distances between the two women.


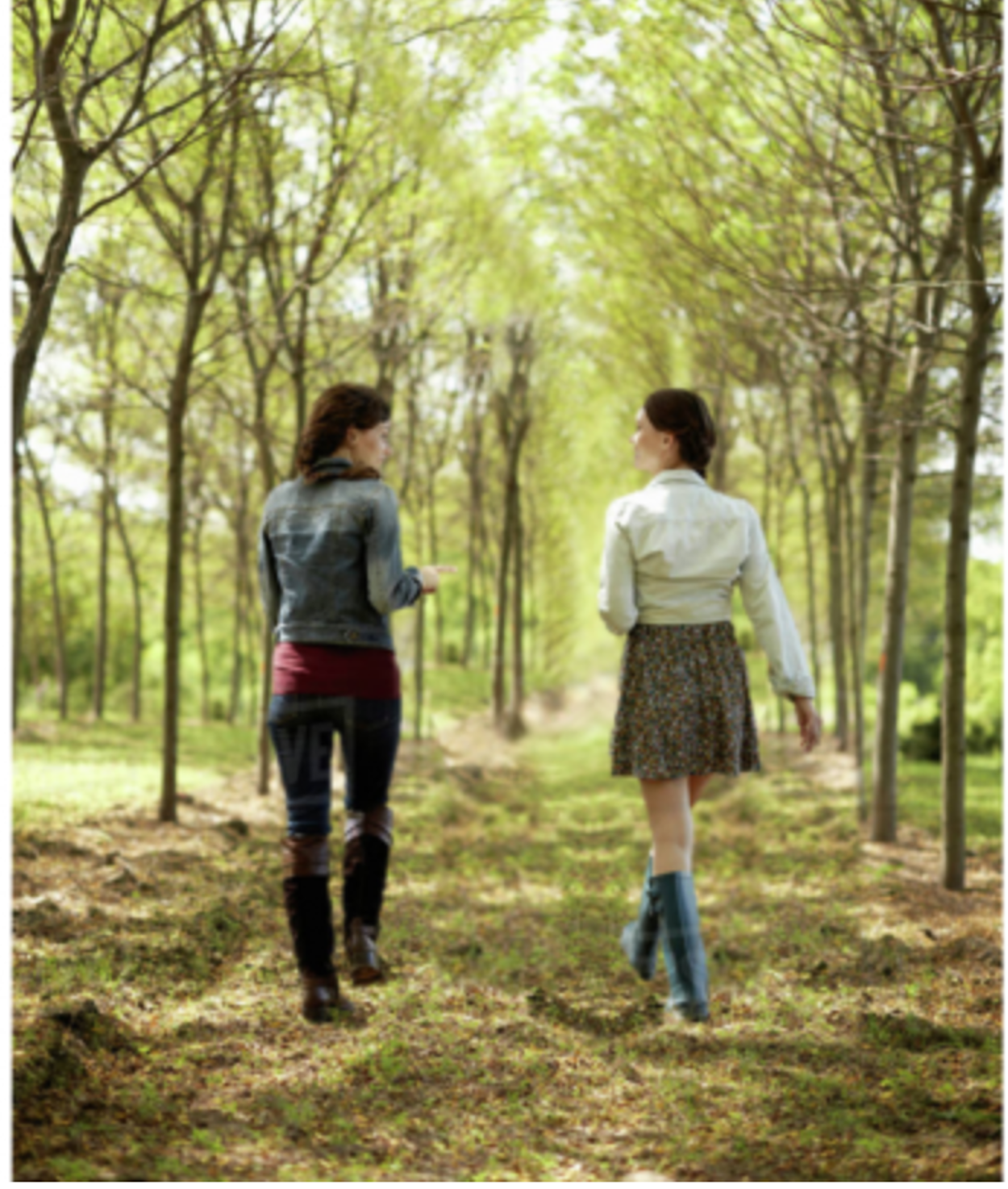


[CrosswalkDistance] “Imagine you are walking across the street and you pass someone walking the other way. How much distance would you leave between you?” This item was measured on an 11-point slider scale, corresponding to images depicting different distances between the two people.


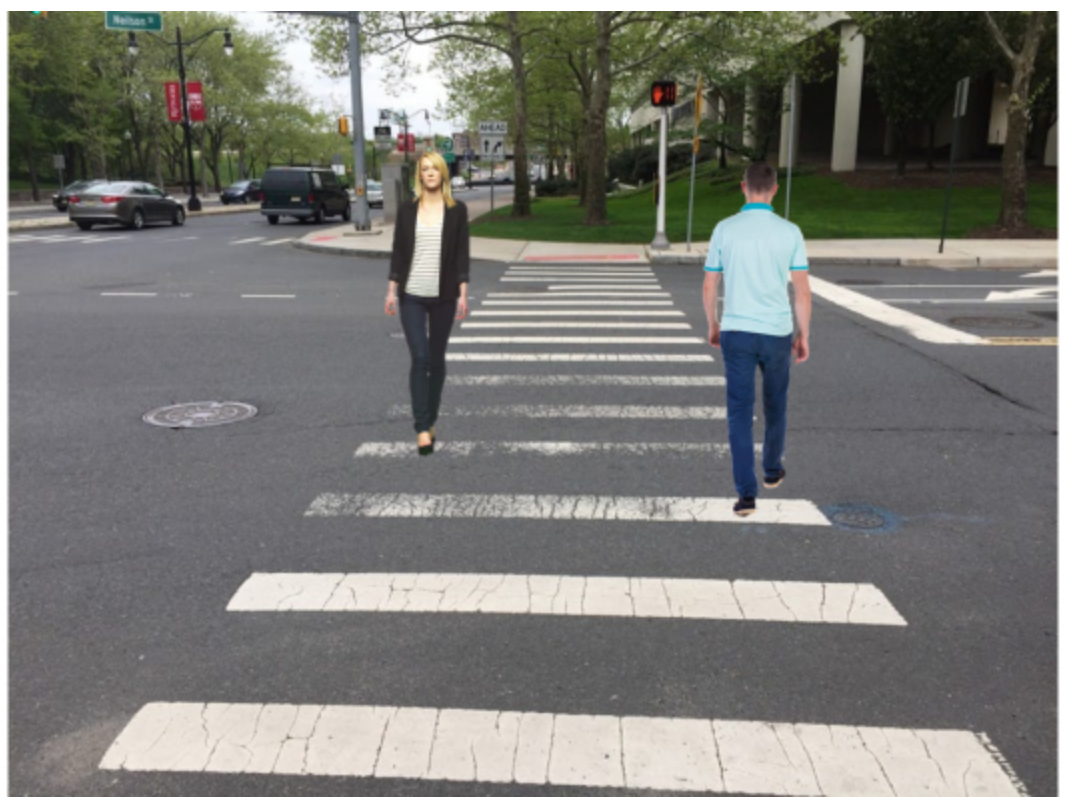


[StandInLineDistance] “Imagine you are standing in line waiting for the bus. How much distance would you want between you and the other people in line?” This item was measured on an 11-point slider scale, corresponding to images depicting different distances between the people.


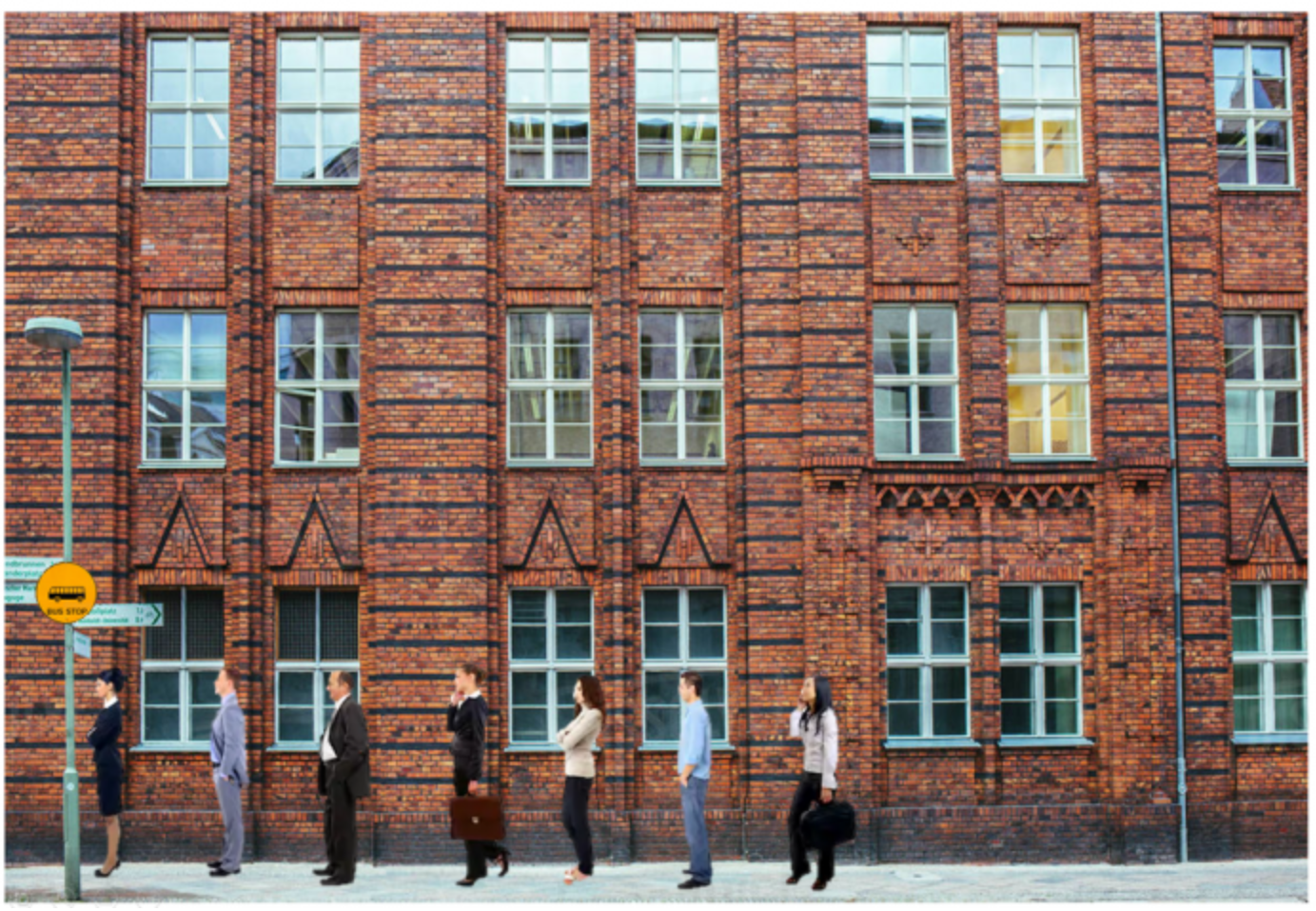


[crowd_path_length] “Imagine that you need to get to the other side of a crowded plaza. What path would you take?” Participants were free to draw any path through the crowd. We then calculated the total distance traveled, in pixels, to determine whether people took a more direct-but-crowded, versus indirect-but-solitary, path. Because of a technical error, the data from this measure were not recorded in Study 3. The red line in the image below illustrates an example response.


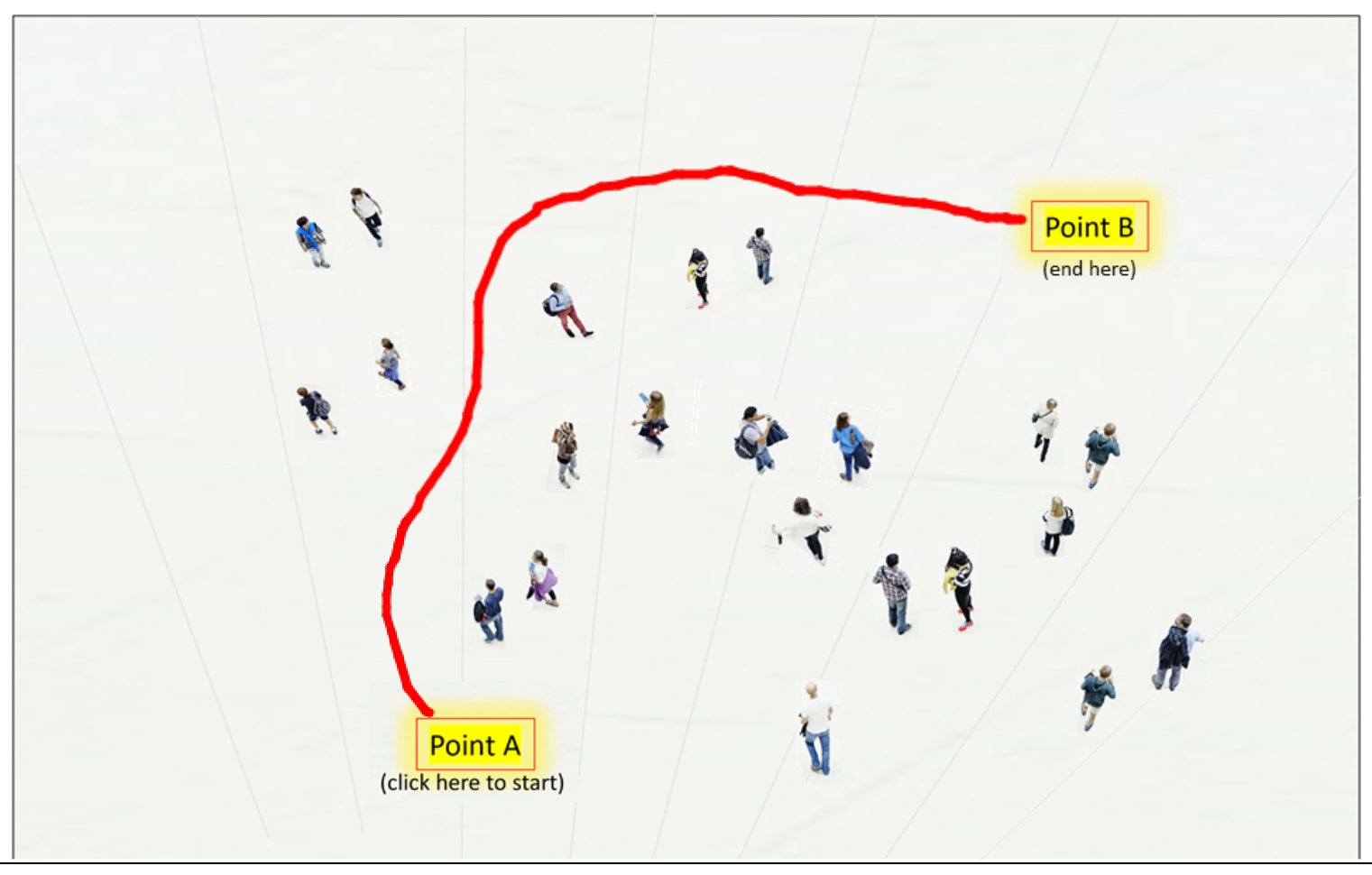


[beach_shortest_distance] “Click the point on the beach where you’d be most likely to lay down your towel.” Participants were free to choose any point on the beach. We then calculated the shortest distance, in pixels, between the point that the participant clicked and the nearest person on the beach.


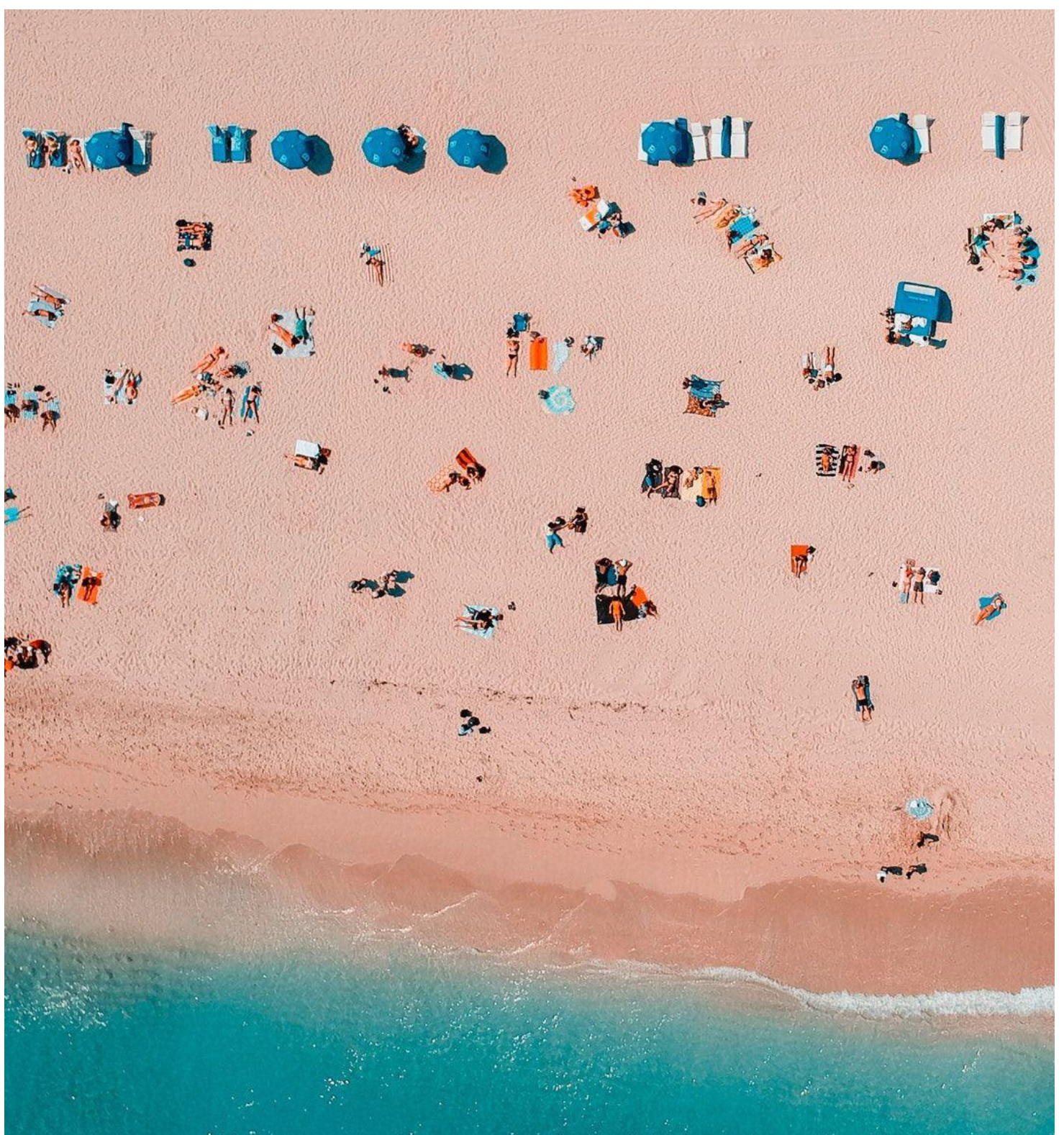


[GroceryStorePeople] “Here is a scene of a very crowded grocery store. Move the slider to “remove” people until you have reached the maximum number of people you would be comfortable having in the grocery store with you.” This item was measured on an 11-point slider scale, corresponding to images depicting different numbers of people.


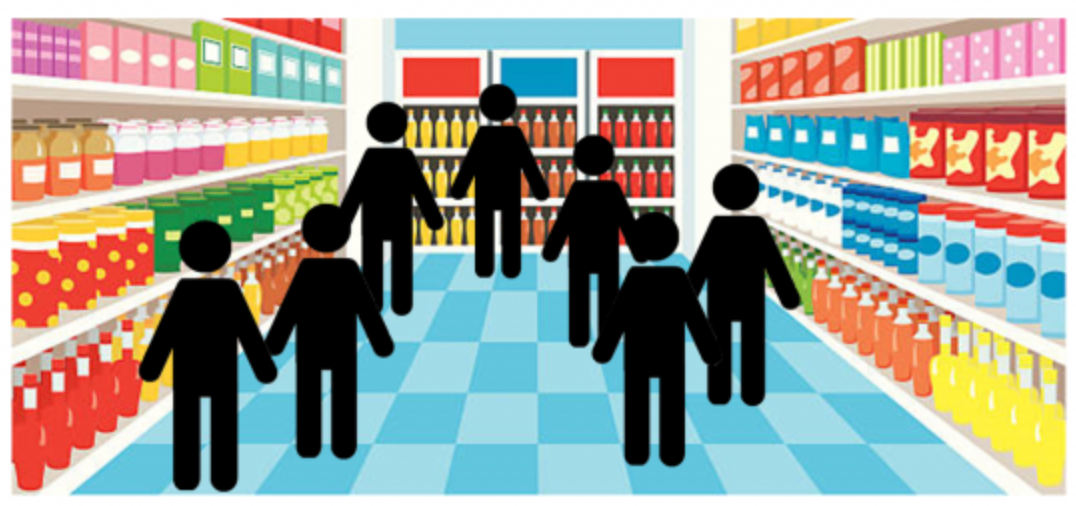


[TodayStreetPaths] “Imagine you are walking down the street when you see a person standing ahead of you at the corner. Do you continue on your path and walk directly by them (A), or do you go out of your way to avoid them (B)?” Responses were made on a 4-point scale: “I would definitely pick A”, “I would probably pick A”, “I would probably pick B”, “I would definitely pick B.”


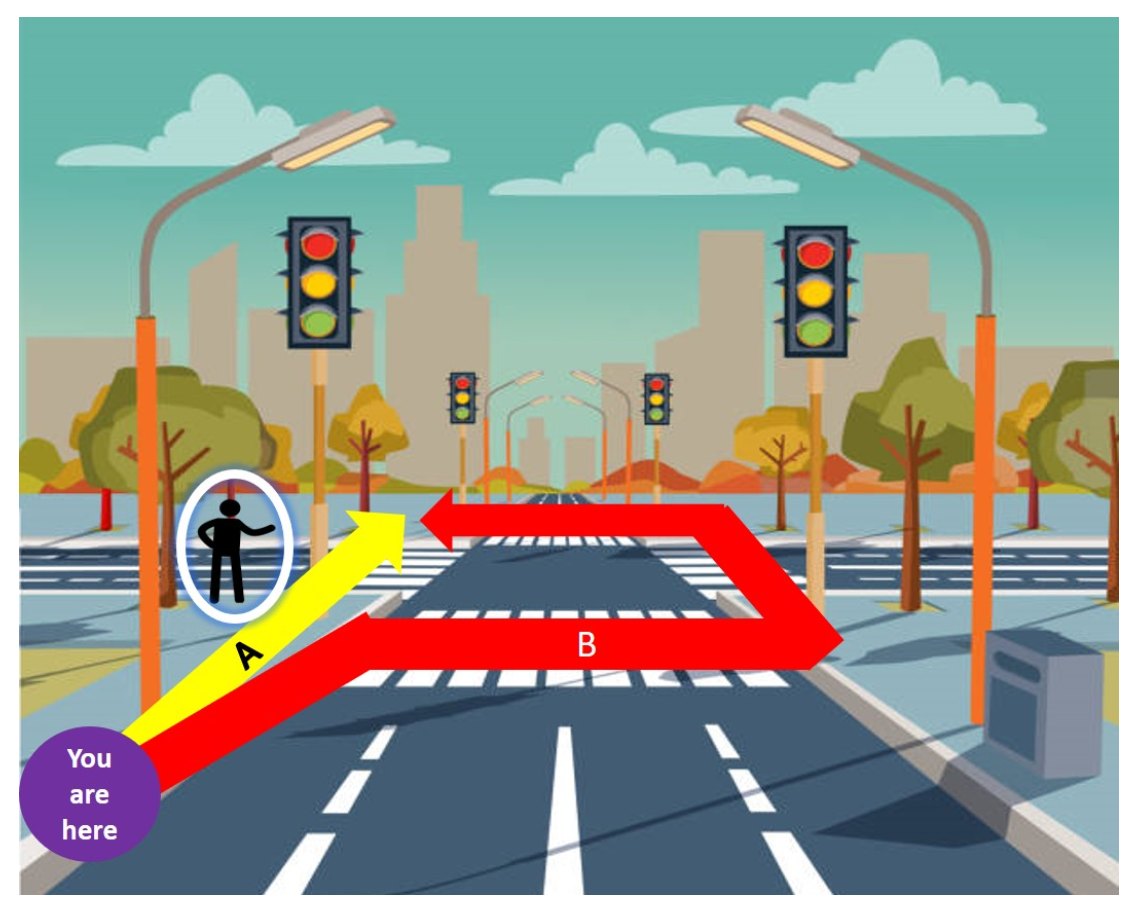


[TodayParkPaths] “Imagine you have to walk through a park to get to a store on the other side, and you have to choose one of two paths. One path through the park is typically quiet and secluded, but it is long (A). The other path typically has more people, but it is much shorter (B). Which path would you most likely choose?” Responses were made on a 4-point scale: “I would definitely pick A”, “I would probably pick A”, “I would probably pick B”, “I would definitely pick B.”


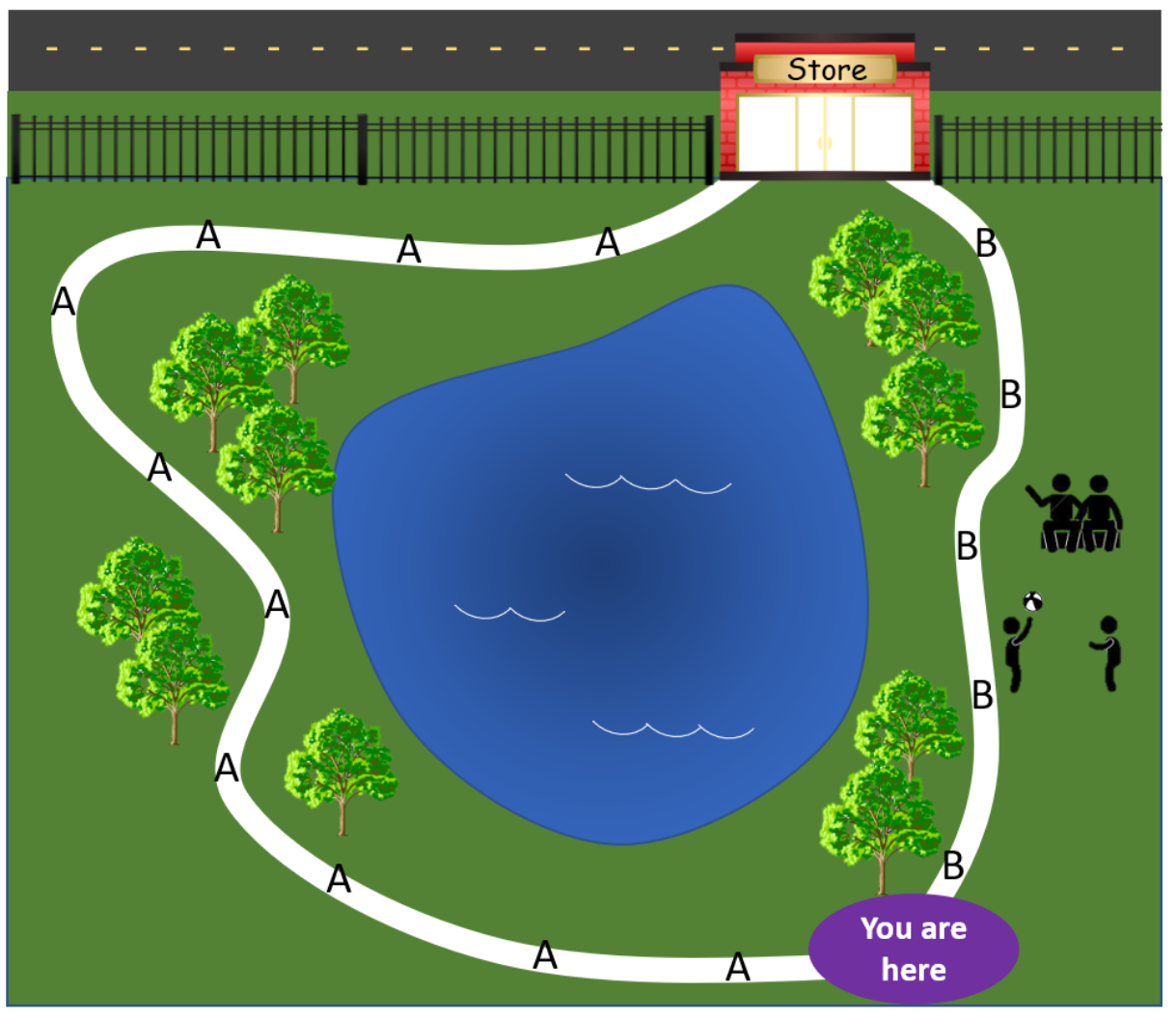


[FutureCoffee] “Imagine you walk into a coffee shop **that is once again open for business**, and you see there are only two open seats (A and B). Which seat would you most likely choose?” Responses were made on a 4-point scale: “I would definitely pick A”, “I would probably pick A”, “I would probably pick B”, “I would definitely pick B.”


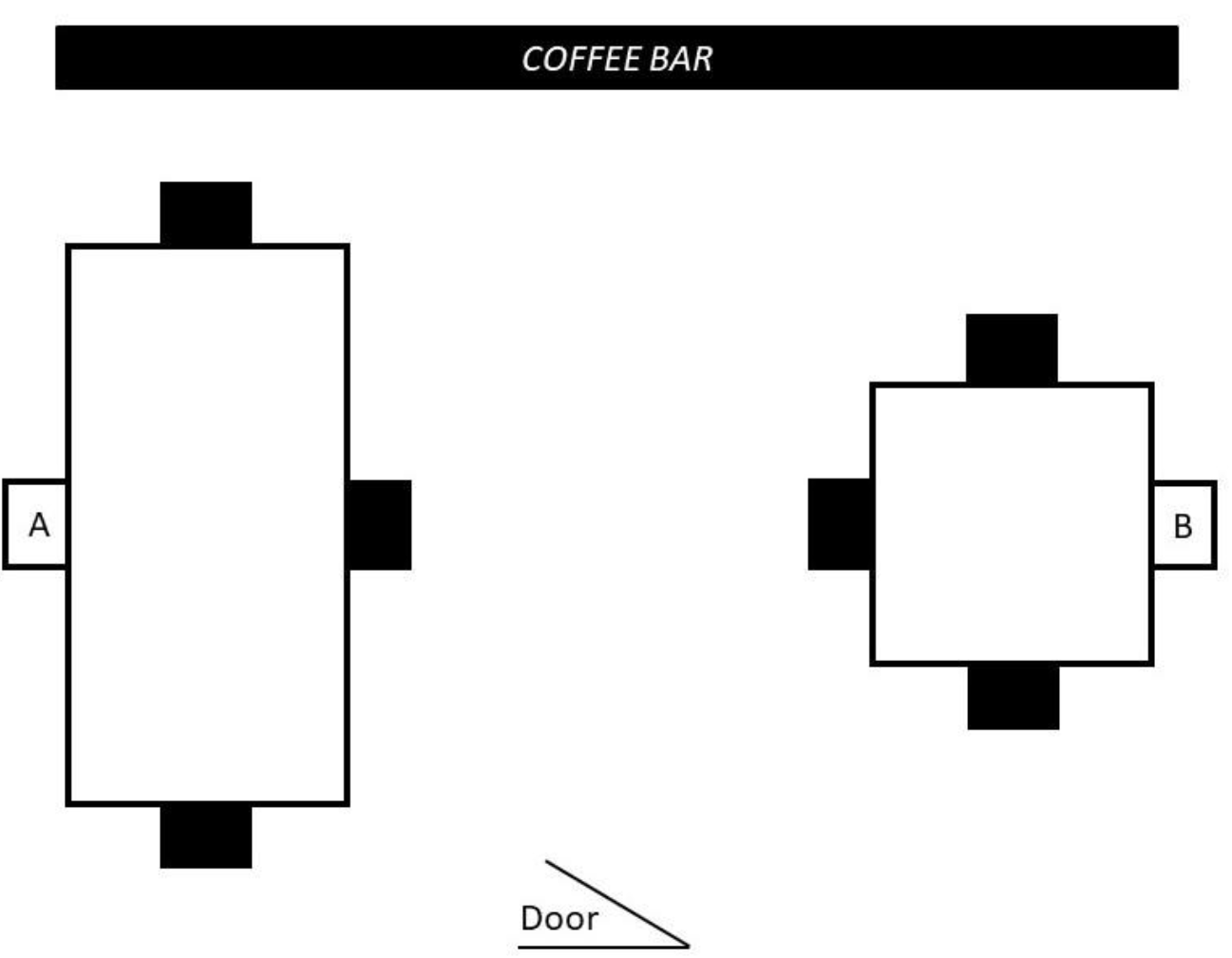


[FutureLibrary] “Imagine you need to return a book at the library **when it has reopened for business**. When you walk in, you see there are two equally direct paths to get to the drop-off site (A and B). Which path would you most likely choose?” Responses were made on a 4-point scale: “I would definitely pick A”, “I would probably pick A”, “I would probably pick B”, “I would definitely pick B.”


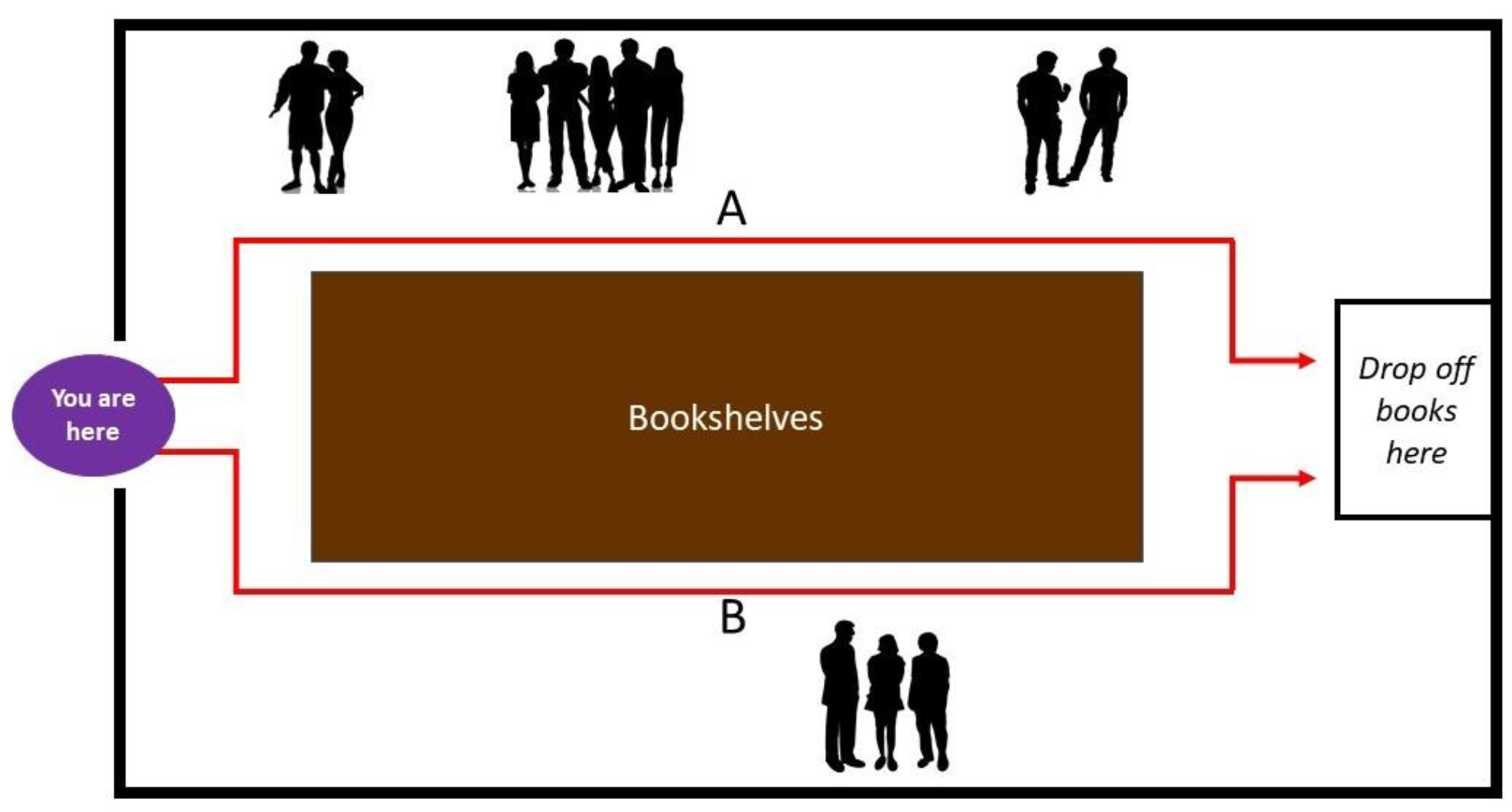


**Scoring of Dependent Measures**

To create our composite pandemic response measure, we first z-scored all items within each study. To give equal weight to each of the three dimensions of pandemic response—attitudes, self-reported behavior, and virtual distancing—we first created separate composite scores for each of these three dimensions by calculating the mean of the standardized items in each category [composite variables are named MeanAttitudes, MeanSelfReportBehavior, and MeanBehavioralDistancing in the dataset]. Finally, we calculated the mean of these three composite scores to create our composite pandemic response measure [MeanOverallCOVIDResponse].

**Attention Check**

To ensure that participants were attending to the study, we included an attention check [attncheck] question: “Bob had a great day. He saw a beautiful butterfly. What did Bob see?” Four response options were provided: “a girl”, “a day”, “a fruit”, “an insect.” Participants who chose any answer other than “an insect” were excluded from our primary analyses.

**Political Orientation**

In all studies, general political orientation [PolOrient] was assessed by a single item: “Please select the scale point that best reflects your political orientation,” measured on a 7-point scale from “Extremely liberal” to “Extremely conservative.” In Study 4, we also assessed social/cultural conservatism [SocOrient] with the item “In terms of social and cultural issues, how liberal or conservative are you?”, and we assessed economic conservatism [EconOrient] with the item “In terms of economic issues, how liberal or conservative are you?” Responses were made on the same 7-point scale from “Extremely liberal” to “Extremely conservative.” Because the pattern of the results with social/cultural conservatism and economic conservatism were very similar to those with general conservatism, we used only the general conservatism item in our analyses in the main text.

**[1] PERSONALITY TRAIT-TYPE FACTORS**

**Conspiratorial Ideation**

*General Conspiratorial Ideation*:

Conspiratorial ideation was measured by the 15-item scale from Brotherton and colleagues (Brotherton, French, & Pickering, 2013), including items such as “Some UFO sightings and rumors are planned or staged in order to distract the public from real alien contact” (α = .95). Each item was measured on a 5-point scale from “Definitely not true” to “Definitely true.” In Study 3, we shortened this scale to ten items (α = .94). [MeanConspiracy]

*COVID-19-Specific Conspiracy Theories:*

Endorsement of conspiracy theories related to COVID-19 was measured by two items: “COVID-19 is intentionally presented as dangerous in order to mislead the public” and “COVID-19 was intentionally brought into the world for dark purposes.” Agreement with each statement was measured on a 7-point scale from “-3 Strongly disagree” to “+3 Strongly agree.” The correlation between these two items was *r* = .77, *p* < .001. [MeanCOVIDConspiracy]

**Disgust Sensitivity**

In Study 1, we measured disgust sensitivity with the 7-item Pathogen Disgust subscale of the Three-Doman Disgust Scale (TDDS; Tybur et al., 2009; [MeanTDDS]), which asks participants to rate how disgusted they would be by experiences such as “Stepping on dog poop” and “Shaking hands with a stranger who has sweaty palms.” Each item was measured on a 7-point scale from “0 Not at all disgusting” to “6 Extremely disgusting” (α = .86).

In Studies 2 and 4, we used the 5-item Contamination subscale of the Disgust Scale-Revised (DSR; Haidt et al. 1994; Olatunji et al., 2007; [MeanDSR]), which depicts several potentially disgusting experiences such as “You take a sip of soda and realize that you drank from the glass that an acquaintance of yours had been drinking from.” Participants were asked to rate how disgusting they would find each experience on a 5-point scale from “0 Not disgusting at all” to “4 Extremely disgusting” or “0 Strongly Disagree” to “4 Strongly Agree” (depending on the nature/phrasing of the item) (α = .70).

The association between TDDS scores and ideology was not significant (β = .08, *t*(116) = 0.84, *p* = .40), although this may partially reflect the small sample to which this scale was administered (*n* = 118). The relation between TDDS scores and pandemic response was of a similar magnitude and also was not significant (β = .08, *t*(116) = 0.91, *p* = .37).

The association between DSR scores and ideology was β = .18, *t*(896) = 5.38, *p* < .001. The relation between DSR scores and pandemic response was β = .24, *t*(898) = 7.44, *p* < .001.

For our analyses in the main text, we z-scored and combined these two measures of disgust sensitivity [MeanDisgust].

**Empathic Concern**

*General Empathic Concern*

In Study 1, we measured empathic concern with three scales:

The 8-item compassionate concern for humanity scale (Sprecher & Fehr, 2005), which includes items such as “I feel selfless caring for most of mankind.” Each item was measured on a 6-point scale from “1 Not at all true of me” to “6 Very true of me” (α = .90). [MeanCompassionateLove]

The 7-item perspective-taking subscale of the Interpersonal Reactivity Index (Davis, 1980), which includes items such as “When I'm upset at someone, I usually try to "put myself in his shoes" for a while.” Each item was measured on a 5-point scale from “1 Does not describe me well” to “5 Describes me very well” (α = .80). [MeanPerspectiveTakingSubscale]

The 7-item empathic concern subscale of the Interpersonal Reactivity Index (Davis, 1980), which includes items such as “I often have tender, concerned feelings for people less fortunate than me.” Each item was measured on a 5-point scale from “1 Does not describe me well” to “5 Describes me very well” (α = .84) [MeanEmpathicConcernSubscale]

In Studies 2 and 4, we removed the Compassionate Concern for Humanity Scale and included only the perspective-taking and empathic concern subscales of the Interpersonal Reactivity Index.

For our analyses in the main text, we z-scored and combined these measures (α of three measures = .76) to create a composite measure of empathic concern [MeanEmpathy].

*COVID-19 Empathy*

COVID-19-specific empathy [MeanCOVIDEmpathy] was assessed by five novel items, each of which was measured on a 6-point scale from “1 Strongly disagree” to “6 Strongly agree”:

“I feel sympathy or compassion toward those affected by COVID-19 coronavirus.”

“I feel it is my personal responsibility to keep others safe from COVID-19 coronavirus.”

“Since the outbreak, I have explicitly avoided spending too much time around the elderly (and/or those with underlying health conditions) because I fear for their health.”

“When I follow social distancing rules, it is primarily because I am concerned about inadvertently infecting others with COVID-19 coronavirus.”

“Many people who are infected with COVID-19 coronavirus got what they deserved.”

In later waves, the third item was replaced by “I am concerned about inadvertently infecting others with COVID-19 coronavirus.” In Studies 2 and 3 we also removed the final two items from this scale to improve reliability. The alpha of the 5-item version was α = .62. The alpha of the revised 3-item version was α = .74.

**[2] ATTITUDE AND BELIEF FACTORS**

**Trust in Government**:

*Trust in the Federal Government*

In Study 1, trust in the federal government was assessed by two items: “On a scale from 0 to 100, what percent of the time do you think you can trust the federal government in Washington?”, measured on a 1-100 slider scale; and “How much confidence do you have in our government leaders?”, measured on a 7-point scale from “0 None at all” to “6 Complete.”

In Study 2, trust in the federal government was assessed with one item: “Generally speaking, how confident are you that the federal government will address the nation’s problems effectively?”, measured on a 7-point scale from, e.g., “0 Not at all” to “6 Very Much.”

*Trust in Trump*

Trust in Trump was assessed in Studies 2 and 4, by two items: “How much do you trust President Trump to lead us effectively through the current COVID-19 crisis?” and “Generally speaking, how much confidence do you have in President Trump?” Both items were measured on 7-point scales from “0 Not at all” to “6 Very Much.”

*Trust in State Governors*

Trust in state governors was assessed by one item: “How much do you trust state Governors to lead us effectively through the current COVID-19 crisis?”, which was measured on a 7-point scale from “0 Not at all” to “6 Very much.”

*Scoring*

For consistency and to facilitate comparison between items, we rescaled the 0-100 scale (Study 1, Item 1) to a 1-7 scale. We then averaged across the appropriate items to create composite scores for each of the three constructs listed above [MeanTrustInFederalGov, MeanTrustInTrump, MeanTrustInStateGovernors]

**Trust in Science:**

We assessed trust in science with two scales:

The 6-item belief in science scale from Farias, Newheiser, Kahane, and de Toledo (2013), which includes items such as “Science is the most efficient means of attaining truth.” Items were measured on a 6-point scale from “1 Strongly disagree” to “6 Strongly agree” (α = .92).

The 11-item trust in science/scientists scale from Nadelson et al. (2014), which includes items such as “We should trust that scientists are being honest in their work.” Items were measured on a 5-point scale from “1 Strongly disagree” to “5 Strongly agree” (α = .91).

For our analyses in the main text, we z-scored and calculated the mean of these two measures to create a composite trust in science score [MeanTrustAndBeliefInScience].

**Perceived Social Norms**

*In-Depth Norms Measure*

In Study 3, we included an in-depth measure of norms that asked participants to complete our behavioral distancing measures as though they were the average American. For the interactive slider social-distancing questions, the pick-a-point-on-the-beach question, and the draw-a-path-through-the-crowd question, participants were simply asked to respond as the average American. For the binary choice social-distancing questions, participants were asked to indicate the proportion of Americans that they believed would choose the option that involved more (versus less) social distancing. These questions were designed so that the total percentage was held constant at 100, and thus the percentage choosing Option A would always be inversely proportional to the percentage choose Option B. However, because of technical errors this automatic scaling feature did not work correctly for a few participants, and the variables were thus not perfectly inversely correlated (correlations were around *r* = .94 for all measures). Accordingly, for our analyses of these items, we calculated the estimated proportion who would choose the more socially distant choice by dividing this percentage by the sum total of the percentages allocated by participants (which could be higher than 100). Higher scores thus indicated greater perceived normativity of the more “socially distant” response.

For our analyses, we z-scored and calculated the mean of these ten items to create a composite measure of perceived social norms [MeanBehavioralDistancingNORM].

*Self-Reported Norm Perceptions*

In Study 4, perceived social norms were measured by a single item: “To what extent do you think that Americans in general are currently following social distancing guidelines?”, measured on a 7-point scale from “1 Not following guidelines at all” to “7 Following guidelines very strictly.” [SelfReport_Norm]

In our main-text analysis assessing the overall relation between ideology and perceptions of social norms, we z-scored and combined these two norms measures into a single index.

**[3] KNOWLEDGE- AND INFORMATION-RELATED FACTORS**

**News Sources:**

Participants’ preferred news sources were assessed with two questions:

“From which of the following news sources have you gotten your news within the past week? Please select all that apply.” Response options were “ABC, CBS, or NBC News”, “CNN”, “Fox News”, “MSNBC”, “NPR”, “National Newspapers/Magazines (e.g., New York Times, Washington Post, Time, Newsweek, USA Today, Wall Street Journal, AP News)”, “Social media (e.g., Facebook, Twitter, Instagram, etc.)”, and “I do not follow the news.”

All participants who did not select “I do not follow the news” then answered the following question: “Which of the following news outlets is your primary source of news? Please select one.” This question had the same response options as above, with the exception of “I do not follow the news.”

As our measure of the ideological slant of one’s primary news source [PrimaryNewsIdeologicalSlant], we recoded responses to the primary news source question such that “CNN”, “MSNBC” and “NPR” were coded as liberal or “-1”; “ABC, CBS, or NBC News” and “I don’t follow the news” were coded as neutral/moderate or “0”; and “Fox News” was coded as conservative or “1.” Because the ideological slant of “National Newspapers/Magazines…” and “Social Media…” were unclear, we omitted these responses from this measure. However, the results of our analyses are very similar if these response options are instead coded as “0.”

**Scientific Literacy:**

We measured scientific literacy with an 11-item true/false quiz (Miller, 1998) that included items such as “Electrons are smaller than atoms” and “Light travels faster than sound.” We then calculated each participant’s total score on this quiz and used this as our measure of scientific literacy [TotalScienceLiteracy].

**Objective COVID-19 Knowledge**:

We measured objective knowledge about COVID-19 with a 13-item true/false quiz:

1. “The seasonal flu vaccine will protect you from COVID-19 / the coronavirus.” (false)

2. “The health effects of COVID-19 / the coronavirus appear to be more severe for people who already have a serious medical condition.” (true)

3. “Antibiotics are an effective treatment for COVID-19 / the coronavirus.” (false)

4. “Packages or letters from China can spread COVID-19 / the coronavirus.” (false)

5. “Spraying chlorine on your body will protect you even if COVID-19 / the coronavirus has already entered your system.” (false)

6. “Regularly rinsing your nose with saline will protect you against COVID-19 / the coronavirus.” (false)

7. “COVID-19 / the coronavirus was deliberately released rather than naturally occurring.” (false)

8. “Some individuals who have COVID-19 / the coronavirus do not show any symptoms.” (true)

9. “Symptoms of COVID-19 / the coronavirus can appear up to 14 days after exposure to the virus.” (true)

10. “Washing your hands with soap and water for at least 20 seconds can reduce the spread of COVID-19 / the coronavirus” (true)

11. “Disinfectant wipes can eliminate COVID-19 / the coronavirus from common household surfaces (e.g. countertops, doorknobs, etc.).” (true)

In Studies 1 and 2, the final two items were:

12. “There is a vaccine to protect against COVID-19 / the coronavirus.” (false)

13. “There is an effective medicine available for treating COVID-19 / the coronavirus.” (false)

In Studies 3 and 4, the final two items were altered somewhat to reflect recent advances in research on the virus:

12. “A vaccine to protect against COVID-19 / the coronavirus is currently available.” (false)

13. “The malaria medication hydroxychloroquine is effective at curing COVID-19 / the coronavirus.” (false)

We calculated each participant’s total score on this quiz and used this as our measure of objective COVID-19 knowledge [TotalCOVIDKnowledge].

**[4] DEMOGRAPHIC FACTORS**

**Income**

Income was assessed with the item: “Please select the annual income of your family to the best of your knowledge”, with eight response options ranging from “Less than $15,000 to “More than $150,000”, as well as “Prefer not to answer.” Participants who selected “Prefer not to answer” were not included in our analyses. [Income]

Income was significantly associated with ideology (β = .05, *t*(3816) = 3.25, *p* = .001). However, income was not associated with response to the pandemic (β = .01, *t*(3820) = 0.78,  *p* = .44).

**Race**

Participants’ race/ethnicity was assessed with the item: “Please select all racial/ethnic categories that apply to you,” with the following response options: “White/Caucasian”, “Black/African-American”, “Hispanic/Latino”, “American Indian or Alaskan Native”, “Asian”, “Native Hawaiian or Pacific Islander”, “Other_____”, and “Prefer not to answer.” Participants were free to select any number of options.

For our analyses in the main text, we focused on whether or not the participant identified as White [RaceWhite]. Conservatism was associated with a greater likelihood of identifying as White (logistic regression, B = .04, χ^2^(1) = 4.18, *p* = .04). Identifying as White (versus not) was associated with less concern about the virus (*t*(2443.93), *p* < .001, unequal variances). This association remained significant after adjusting for participants’ ideology (*F*(1,3878) = 13.43, *p* < .001). However, identifying as White did not account for a significant portion of the ideological gap: the association between ideology and pandemic response was not meaningfully attenuated when race (White versus non-White) was added to the model (β reduction = .002).

**Religion**

Religion was assessed only in Study 2, to a total sample of 1,823 people. It was measured with the item “What religion do you belong to?”, with the following response options “Atheist or Agnostic”, “Christianity – Catholicism”, “Christianity – Protestantism”, “Judaism”, “Islam”, Hinduism”, “Buddhism”, “Other____” and “Christianity – Other.” In our analyses, we focused on whether or not participants identified as Christian [ReligionChristian].

Greater conservatism was associated with a higher likelihood of identifying as Christian (logistic regression, B = .44, χ^2^(1) = 206.63, *p* < .001). Identifying as Christian was associated with greater concern about the virus (*t*(1821) = 4.04, *p* < .001). However, identifying as Christian was no longer a significant predictor of pandemic response after adjusting for ideology (*F*(1,1819) = 0.59, *p* = .44).

**Religiosity**

Religiosity was assessed only in Study 2, to a sample of 1,823 people. It was measured with the item “How religious are you?”, rated on a 5-point scale from “Not at all” to “Extremely.” [Religiosity]

Conservatism was associated with greater religiosity (β = .37, *t*(1820) = 17.23, *p* < .001). Religiosity was associated with less concern about the pandemic (β = -.10, *t*(1821) = 4.14,  *p* < .001). However, religiosity was no longer a significant predictor of pandemic response after controlling for ideology (β = .02, *p* = .32).

**Education**

Education was assessed with the item “What is your highest level of education?”, with the options “Less than high school graduate”, “High school graduate or general education diploma”, “Some college”, “College degree”, “Post-graduate degree”, and “Prefer not to answer.” Participants who selected “Prefer not to answer” were excluded from analyses. [Education]

Education was not associated with political ideology (β = .009, *t*(3864) = 0.56, *p* = .58). However, education was associated with responses to the pandemic, such that more educated individuals expressed greater concern about the virus (β = .08, *t*(3868) = 5.13, *p* < .001).

**[5] VULNERABILITY FACTORS**

**Preexisting Conditions**

Whether and to what degree a participant had preexisting conditions that made them more vulnerable to the virus was measured by the item: “Consider your personal health prior to the outbreak of the COVID-19 virus. Would you have described yourself as having any preexisting conditions that made you more vulnerable to the disease?” Participants responded on a 5-point scale from “Definitely not” to “Definitely yes.” [PreexistingCondition]

Greater conservatism was weakly, although significantly, associated with preexisting conditions (β = .06, *t*(3376) = 3.22,  *p* = .001). Having preexisting conditions was associated with greater concern about the virus (β = .16, *t*(3379) = 9.46,  *p* < .001).

**Perceived Vulnerability to Disease**

Participants’ subjective sense of vulnerability to disease was measured by the 15-item Perceived Vulnerability to Disease Scale (Duncan, Schaller, & Park, 2009), including items such as “I am more likely than the people around me to catch an infectious disease.” Agreement with each item was measured on a 5-point scale from “0 Strongly Disagree” to “4 Strongly Agree” (α = .75). [MeanPVD]

Perceived vulnerability to disease was not associated with ideology (β = .05, *t*(1013) = 1.43,  *p* = .15).

**Age**

Age was measured with the item “How old are you?”, followed by an open-response text entry box. [Age]

Political ideology was associated with age: β = .08, *t*(3877) = 5.22,  *p* < .001. Age was weakly, although significantly, associated with responses to the pandemic (β = .06, *t*(3881) = 3.99,  *p* < .001).

**[6] NEGATIVE-IMPACT FACTORS**

**Negative Economic Consequences**

We assessed participants’ subjective sense of having suffered economically from COVID-19 with the item “To what degree have you and your immediately family personally suffered negative economic/financial consequences (e.g., laid-off, lost wages) because of the COVID-19 / coronavirus restrictions?”, measured on a 7-point scale from “1 Not at all” to “7 Very much.” [OwnEconConsequences]

Conservatives were somewhat more likely to report having experienced negative economic consequences from the pandemic (β = .09, *t*(3649) = 5.47,  *p* < .001). However, suffering negative economic consequences was associated with greater concern about the virus (β = .09, *t*(3653) = 5.22,  *p* < .001).

**Contracting COVID-19**

To assess whether participants had contracted COVID-19, we first asked whether they had been tested for COVID-19: “Have you been tested for COVID-19 / the coronavirus?” [COVIDTested]. Depending on their response, they were then asked one of two questions. If they reported having been tested, they were asked “Did the test show that you have/had COVID-19 / the coronavirus?” If they reported having not been tested, they were asked “Even though you may not have been tested, do you believe that you may currently have (or may previously have had) COVID-19 / the coronavirus?” In Study 1, the questions about testing and whether the participant had contracted COVID-19 were combined into a single question with four response options.

For the purpose of analyses, we coded individuals who had been tested and had the virus, as well as those who believed they had the virus but had not been tested, as a “yes” response. All others were coded as “no” [HaveCOVID]. However, the association that we observed with ideology, with conservatives being more likely to have contracted COVID-19, remains significant if responses are restricted only to individuals (*n* = 648) who had been tested for the virus (B = -.14, χ^2^(1) = 6.12, *p* = .01).

Interestingly, having the virus (versus not) was associated with *less* concern about the pandemic (*t*(505.40) = 2.09, *p* = .04, unequal variances), although this association was no longer significant after controlling for ideology (*p* = .07).

**Personal and Familial Job Loss**

In Studies 1 and 2, we assessed personal and familial job loss with the item “Have you and/or any member of your immediately family lost a job or been furloughed due to COVID-19 / the coronavirus?”, which included the response options “I have” and “Someone in my immediate family has.” [LostJobFamilyMember] In Studies 3 and 4, we assessed only personal job loss, using the item “What is your current employment status?”, which included the response option “I lost my job or have been furloughed due to COVID-19 / the coronavirus.” We recoded the two questions regarding personal job loss into a single binary index of whether the participant had personally lost their job [LostJobSelf].

Having personally lost one’s job was associated with greater concern about the virus (*t*(3600) = 2.13, *p* = .03), although having a family member who had lost a job was not (*p* = .61). Liberals and conservatives did not differ in their likelihood of having personally lost their job (*p* = .42) or having a family member who had lost a job (*p* = .52).

**[7] ENVIRONMENTAL FACTORS**

The majority of the environmental factors we examined were assessed at the county-level. To obtain participants’ home counties, we reverse geocoded the zip codes that they provided. Because zip codes sometimes bridge multiple counties, there is some inaccuracy in this approach. To counter this inaccuracy, we also collected participants’ zip codes from their IP addresses, and then reverse geocoded these captured zip codes to provide a second geolocation point. For each of our environmental factors, we calculated one value based on the participant’s reported zip code, and a second based on their captured zip code. We then calculated the mean of these two values and used this combined index in all analyses. Validation checks suggested that this composite measure was more accurate (e.g., the combined measure of county-level conservatism was more strongly associated with participants’ own political ideologies than either the captured or reported locations alone).

**Objective COVID-19 Prevalence and Death Rates**

We retrieved objective COVID-19 infection and death rates using the data collected by Dong, Du, and Gardner (2020) from Johns Hopkins University. The raw data are available here: <https://github.com/CSSEGISandData/COVID-19>. For our analyses, we used the current per-capita county-level infection and death rates on the day that the survey was conducted. We log-transformed these values to account for the extreme positive skew. We then created a composite score by calculating the average of the z-scored, log-transformed death rate and confirmed cases per capita. We used this variable [MeanInfectionPerCapita] in all analyses.

The relation between ideology and infection/death rates was β = -.08, *t*(3878) = 5.03,  *p* < .001

**County-Level Conservatism**

Our index of county-level conservatism was the ratio of votes cast for the Republican versus Democratic candidate in the 2016 election. We calculated these values using the following formula: Republican Votes / Republican Votes + Democratic Votes [RepublicanVote2016].

The relation between ideology and county-level conservatism was β = .14, *t*(3879) = 8.50,  *p* < .001.

**Median Income**

We collected information on median income from the U.S. Census Bureau [MedianIncome]. The values we used were based on data collected in 2014.

Median income was weakly related to pandemic response (β = .05, *t*(3803) = 3.35, *p* = .001), such that people from higher-income areas exhibited greater concern about the virus. This relation also remained nearly identical when controlling for participants’ personal household income (β = .06, *t*(3740) = 3.29, *p* = .001). However, median income was not related to ideology (β = -.02, *t*(3799) = 1.07,  *p* = .29).

**Income Inequality**

As our measure of income inequality, we examined county-level Gini coefficients. The Gini coefficient is a measure of statistical dispersion that indexes the degree of income inequality in an area, ranging from 0, representing perfect equality, to 1, representing perfect inequality (e.g., where all the wealth is held by a single individual). The values we used were based on data collected in 2015 [GiniCoefficient].

Gini coefficients were significantly, although weakly, related to political ideology (β = -.06, *t*(3761) = 3.82,  *p* < .001), such that more conservative individuals tended to live in less economically unequal counties. However, income inequality was not significantly related to responses to the pandemic (β = .03, *t*(3765) = 1.54,  *p* = .12).

**Racial Diversity**

We collected statistics on county-level racial diversity from the U.S. Census Bureau. We focused primarily on the percentage of White (versus non-White) residents in a given county [PercentWhite]. The values we used were based on data collected in 2014.

Ideology was associated with racial diversity (β = .06, *t*(3879) = 3.44, *p* = .001), such that more conservative participants tended to live in areas with more White (versus non-White) residents. A greater White (versus non-White) population was weakly associated with less concern about the virus (β = -.05, *t*(3883) = 2.97, *p* = .003), even when adjusting for participants’ ideology (β = -.03, *t*(3878) = 2.08,  *p* = .04). However, these effects were generally small, and county-level racial diversity accounted for only an extremely small (albeit statistically significant) portion of the ideological gap (indirect effect: β = -.002, 95% CI[-.004,-.0002]).

**Population Density**

We collected information on county-level population density from the U.S. Census Bureau. We operationalized population density as the number of residents per square mile [PersonsPerSquareMile]. The values that we used were based on data collected in 2010.

Ideology was associated with population density (β = -.05, *t*(3877) = 2.95, *p* = .003), such that more conservative participants tended to live in areas with fewer residents per square mile. Greater population density was weakly associated with greater concern about the virus (β = .04, *t*(3881) = 2.32, *p* = .02). However, this association was no longer significant when adjusting for participants’ own ideology (β = .02, *t*(3876) = 1.54, *p* = .12). Further, population density did not significantly statistically mediate the ideological gap in pandemic response (indirect effect: β = -.001, 95% CI[-.002, .00001]).

**Age Distribution**

We collected information about county-level age distributions from the U.S. Census Bureau. We focused on the percentage of residents in each participant’s county that were 65 and older [Percent65AndOver]. The values we used were based on data collected in 2010.

Ideology was not associated with the percentage of the population that was age 65 and older (β = .02, *t*(3824) = 1.30, *p* = .20). The percentage that was 65 and older was also not associated with responses to the pandemic either without (β = -.02, *t*(3828) = 1.27, *p* = .21) or with (β = -.03, *t*(3825) = 1.61, *p* = .11) participants’ own age as a covariate.

**State Governor Political Party**

We also assessed the relation between pandemic response and the political party of participants’ state governors [GovernorPartyNumeric]. We collected information on each governor’s political party from the usa.gov website.

More conservative participants were more likely to live in areas with Republican (versus Democratic) governors (logistic regression, B = .06, χ^2^(1) = 10.23, *p* = .001). The political party of participants’ state governors (Democrat versus Republican) was associated with pandemic response, such that participants living in areas with Democratic governors were more concerned about the virus (*t*(3410.65) = 3.67, *p* < .001, unequal variances). This association also remained significant when controlling for participants’ own political ideology (*F*(1,3864) = 8.49, *p* = .004). However, the political party of one’s state governor did not account for a significant portion of the ideological gap: the association between ideology and pandemic response was not meaningfully attenuated when governors’ political party was added to the model (β reduction = .003).

**Reopening Status**

In all studies after Study 1 (during which most of the U.S. was still under strict lockdown orders), we assessed whether participants’ local communities had begun to reopen, using the item “Has the particular area in which you live relaxed restrictions and allowed restaurants, stores, libraries, playgrounds, and the like to reopen?” Participants in Study 2 were provided a binary yes/no response option [ReopenedBinary]. Participants in Studies 3 and 4 were provided an additional response option indicating partial reopening: “Partially; my area is beginning to reopen.” We observed stark differences in reopening status across our earlier and later studies. In Study 2, only 37% of participants indicated that their areas had reopened. In Study 4, 95% of participants indicated a full or partial reopening. Because of the lack of variance that a binary no/yes categorization provides in our later studies, in our analyses we focused on the 3-response-option item from Studies 3 and 4 [ReopenedThreeOption].

More conservative participants tended to live in areas that had more fully reopened (β = .06, *t*(1560) = 2.52, *p* = .01). Living in areas that had more fully reopened was associated with less concern about the pandemic (β = .10, *t*(1562) = 3.84, *p* < .001). However, reopening status explained only a very small, although statistically significant portion of the ideological gap in pandemic response (indirect effect: β = .006, 95% CI[.0008,.01]).

**References Not Listed in the Main Text**

Dong, E., Du, H., & Gardner, L. (2020). An interactive web-based dashboard to track COVID-19 in real time. *The Lancet infectious diseases*, *20*(5), 533-534.

Duncan, L. A., Schaller, M., & Park, J. H. (2009). Perceived vulnerability to disease: Development and validation of a 15-item self-report instrument. *Personality and Individual differences*, *47*(6), 541-546.
